# Adipocyte-derived extracellular vesicles: bridging the communications between obesity and tumor microenvironment

**DOI:** 10.1007/s12672-023-00704-4

**Published:** 2023-06-08

**Authors:** Chuan Zhou, Yu-Qian Huang, Ming-Xu Da, Wei-Lin Jin, Feng-Hai Zhou

**Affiliations:** 1 The First Clinical Medical College, Lanzhou University, Lanzhou, 730000 People’s Republic of China; 2 Key Laboratory of Molecular Diagnostics and Precision Medicine for Surgical Oncology in Gansu Province, Gansu Provincial Hospital, Lanzhou, 730000 People’s Republic of China; 3 Department of Center of Medical Cosmetology, Chengdu Second People’s Hospital, Chengdu, 610017 People’s Republic of China; 4 Department of Surgical Oncology, Gansu Provincial Hospital, Lanzhou, 730000 People’s Republic of China; 5 Institute of Cancer Neuroscience, Medical Frontier Innovation Research Center, The First Hospital of Lanzhou University, Lanzhou, 730000 People’s Republic of China; 6 Department of Urology, Gansu Provincial Hospital, Lanzhou, 730000 People’s Republic of China

**Keywords:** Extracellular vesicles, Obesity, Adipocyte, Tumor microenvironment, Cancer

## Abstract

By the year 2035 more than 4 billion people might be affected by obesity and being overweight. Adipocyte-derived Extracellular Vesicles (ADEVs/ADEV-singular) are essential for communication between the tumor microenvironment (TME) and obesity, emerging as a prominent mechanism of tumor progression. Adipose tissue (AT) becomes hypertrophic and hyperplastic in an obese state resulting in insulin resistance in the body. This modifies the energy supply to tumor cells and simultaneously stimulates the production of pro-inflammatory adipokines. In addition, obese AT has a dysregulated cargo content of discharged ADEVs, leading to elevated amounts of pro-inflammatory proteins, fatty acids, and carcinogenic microRNAs. ADEVs are strongly associated with hallmarks of cancer (proliferation and resistance to cell death, angiogenesis, invasion, metastasis, immunological response) and may be useful as biomarkers and antitumor therapy strategy. Given the present developments in obesity and cancer-related research, we conclude by outlining significant challenges and significant advances that must be addressed expeditiously to promote ADEVs research and clinical applications.

## Introduction

Obesity and overweight are risk factors closely linked to cancer incidence, survival, and mortality among patients. Obesity prevalence of obesity has reached epidemic proportions, and continues to trend alarmingly upward. According to data from the World Health Organization (WHO), Worldwide obesity has nearly tripled since 1975 with 39% of persons over the age of 18 being overweight or obese [1]. The Global Obesity Observatory’s latest projections of global overweight or obesity (body mass index BMI ≥ 25 kg/m^2^) show that among persons aged > 5 years, the number of obese or overweight people will reach 4 billion by 2035, increasing from 38% of the population in 2020 to 50%. It is anticipated that the prevalence of obesity (BMI 30 kg/m^2^) would increase from 14% in 2020 to 24% in 2035, reaching approximately 2 billion people by 2035 [2]. Every 5 kg/m^2^ rise over the norm of an individual’s BMI, increases the risk of uterine cancer by 62%, gallbladder cancer by 31%, kidney cancer by 25%, cervical cancer by 10%, and thyroid cancer and leukemia by around 9% [3, 4]. BMI is also associated with an increased risk of liver, colorectal, ovarian, and postmenopausal breast cancers [5]. Dietary factors can also modulate tumor initiation and growth [6]. Increased chronic inflammation and alterations in immune cell communities have been identified as a major connection between obesity and malignancies [7]. Thus, it is crucial to understand the influence of tumors on surrounding cells in an obese state.

The TME is a highly complex local environment made up of multiple cells which include primary tumor cells, numerous stromal cells (including endothelium, and fibroblasts), various immune cells, adipocytes, extracellular fluids. It is also related with several physicochemical factors, most notably hypoxia and decrease in pH [8]. This complex internal milieu creates a favorable microenvironment for tumor development and metastasis by allowing numerous cellular and stromal components to interact and influence one another. A low-oxygen microenvironment develops in the tumor tissue as a result of the rapid multiplication of tumor cells, which in turn increases the tumor’s internal demand for nutrients and oxygen. Furthermore, tumor cells constantly modify their gene expression, and metabolic reprogramming, to adapt to this hypoxic microenvironment of the tumor [8–10].

Adipocytes are lipid-rich, intensely secretory cells that facilitate the storage of long-chain fatty acids via lipid droplets composed of triacylglycerol and cholesterol esters. Adipocytes are typically regarded as energy storage cells. However, in recent years there has been increase in interest in the studies of the chemicals present in secretome of AT and adipocyte, which regulate inflammation, metastasis, and metabolic remodeling [11]. This review will focus on the function of extracellular vesicles (EVs/EV-singular) released by several types of adipocytes in the growth of tumors and the metabolism of obesity.

Expanding research on adiposity has shown that the function of adipocytes in the human body is controlled by the location, distribution, and kind of microenvironment in which they reside. AT based on the adipocyte composition can be divided into three types: white, beige, and brown [12]. EVs secreted specifically from brown AT have garnered substantial attention in recent years. This tissue type is a main adaptive thermogenic site, with evidence showing that its capacity to use glucose and lipids for thermogenesis is significant to the prevention of obesity and metabolic disorders [13]. There are geographical, morphological, functional, and cancer susceptibility dissimilarities between adipose-derived stem cells (ADSCs), cancer-associated adipocytes (CAAs), tumor-stromal adipocytes (TSAs), and normal adipocytes in other sections of the body [14–16] (Table 1).

**Table 1:** Comparison of various types of adipocytes

	Normal adipocyte	ObAs	CAAs	TSAs	ADSCs
Definition	Non-tumor related and normal adipocytes	Hyperplastic and tightly distributed adipocyte	Invading abnormal adipocytes at the entrance of the tumor	One of the stromal cells in the tumor microenvironment	Mesenchymal stem cells with multiple differentiation potential
Morphology	Normal size, round shape, rich in lipid droplets that occupy 90% of the cell volume	Larger size, hypertrophic, round shape, spherical, lipid-rich	Larger size, dedifferentiated condition, fibroblast-like phenotype, decreased lipid content, and smaller size	Smaller size, spindle or ellipsoidal, decreased lipid contends	Smaller size, undifferentiated, needle-shaped, and devoid of lipid droplets
Function	Normal secretion function, possesses energy storage capacity, and maintains energy balance	Induces insulin resistance and dysfunctional insulin secretion inside the body	Various abnormally produced cytokines, adipokines, lipid metabolites, and EVs contribute to the malignant growth of tumors	Important stromal components interact with tumor cells and other stromal cells via releasing various adipokines, lipid metabolites, and exosomes	Capability for multilineage differentiation and self-renewal
The main impact of EVs	Transmits normal metabolic signals and maintains cellular communication	Hepatic AKT phosphorylation, Metabolic reprogramming, promoting cancer growth	Alter the metabolic and immunological state of TME, encourage the proliferation, migration, invasion, and metastasis of tumor cells	Interact with other stromal cells, contributes to the development of the TME	Promote tissue repair and regeneration

*ObA* obese adipocytes, *CAAs* cancer-associated adipocytes, *TSAs* tumor-stromal adipocytes, *ADSCs* adipose-derived stem cells, *TME* tumor microenvironment, *EVs* extracellular vesicles, *AKT* AKT signaling pathway

New evidence reveals that Adipocyte-derived extracellular vesicles (ADEVs) constitute a large fraction of the AT secretome and may play a role in the etiology of obesity-related metabolic problems [17, 18]. The transfer of biological mediators via EVs is quite a particular and highly regulated transport mechanism [19, 20]. To promote tumor growth, metastasis, and therapeutic resistance, EVs boost cell–cell contact inside the TME [21–23]. According to studies, ADEVs or exosomes are critical in triggering the etiology of numerous illnesses in obese individuals. Studies have begun to focus on the modification of the contents of EVs from dysfunctional adipocytes such as miRNA and proteins, and how they impact receiving cells. EVs carry biological cargo, thus a thorough analysis of how they affect recipient cells or how they contribute to the development of illness can provide a comprehensive picture that represents the pathophysiological state.

This review aims to highlight the most current studies on the pathogenic consequences of ADEVs in obesity, as well as how hypoxic TME affects the composition, production, and release of adipose tissue-derived EVs (ATEVs)and ADEVs. The study also gives an outline of the current level of research and use of EV-based therapies.

## Overview of extracellular vesicles

EVs are vesicles with a lipid bilayer membrane that originate from the cell membrane by either direct budding or the fusion of an endosomal-derived multivesicular body with the cell surface [24] (Table 2). Most studies on EVs have focused on their role as signaling carriers in cellular homeostasis and response to changes in pathophysiology, as well as their ability to transport nucleic acids, lipids, and proteins across cells [25]. In previous studies, microsomes and exosomes are the two primary groups of EVs that have emerged from the extensive naming scheme that takes into account the EVs’ cellular origin, size, shape, and payload [25]. Multivesicular endosomes (MVEs) are intraluminal vesicles that fuse with the cell surface to release their contents. Exosomes are generated when the endosomal membrane buds inward during MVE maturation [26, 27]. Microvesicles are created when the plasma membrane buds and splits apart, releasing intracellular contents into the extracellular environment. The diameter range of microvesicles is larger than the diameter range of exosomes, and the size range partially overlaps, between approximately 50 nm and 1000 nm [28]. Since the titles were frequently used interchangeably, it was not always apparent which kind of EV was being studied in the first articles [29]. In addition, EVs are often examined after being isolated from biofluids or cell culture-conditioned media, which include mixtures of EVs from different membrane or organelle origins [29]. Now that EVs smaller than 150 nm in diameter have been proven to both bud from the plasma membrane and be generated inside endosomes as intraluminal vesicles, it is obvious that not all tiny EVs are exosomes. In light of these problems, the EV sector has been working to describe vesicles in terms of their physical properties rather than their biogenesis method [24, 30]. The International Society for Extracellular Vesicles (ISEV) updated minimal experimental standards and recommendations for EV research in 2018 and 2021 [24, 31]. In this review, publications are evaluated according to those guidelines. Extracellular Vesicle is used to refer to exosomes, microvesicles, and other forms of EVs throughout the study.

**Table 2:** EV classification based on size and origin

EVs subtype	Size (nm in diameter)	Origin	Discharge method	Markers	Ref.
Exosomes	30–150	ILVs	MVB	CD9, CD63, CD81, HSP70, TSG101, flotillin-1, ALIX	[69]
Ectosomes	100–1000	Plasma membrane	The severance of protrusions or bumps on the surface of the cell membrane	Selectins, integrins, CD40, ARF6	[203, 204]
Apoptotic bodies	50–5000	Plasma membrane (during apoptosis)	Produced by orderly dividing apoptotic cells	C1q, ICAM-3, clathrin, calreticulin, CD44v6	[205]
Migrasomes	500–3000	Retraction fibres (during migration)	Formed by separation of retraction fibers	NDST1, PIGK, CPQ, EOGT	[206]
Mitosomes	N.D	Separation from migrasomes	Formed by separation of migrasomes	N.D	[207]
Exomeres	< 50	Separation from exosomes	MVB	Hsp90-b	[208]
Supermeres	Around half the volume of exomere	Separation from exomeres	MVB	TGFBi	[209]

*ILVs* intraluminal vescicles, *MVB* multivesicular body

EVs have become a crucial new method of cellular communication and a pathway for the exchange of bioactive chemicals [25]. Emerging evidence suggests that EVs play a significant role in the interaction between adipocytes and tumor cells, complementing soluble substances. ADEVs increase the aggressiveness of melanoma cells by transporting enzymes and lipid substrates; this effect is increased in obese people [32]. According to breast cancer research, human adipose-derived mesenchymal stem cells (ADMSCs) proliferate and migrate when exposed to EVs [33]. Additionally, recent research showed that adipose tissue from obese people secretes chemicals and EVs with pro-tumorigenic properties that can promote BC cell malignancy by activating the PI3K/AKT and ERK/MAPK pathways [34].

## The impact of obesity on ADEVs

**Fig. 1:**
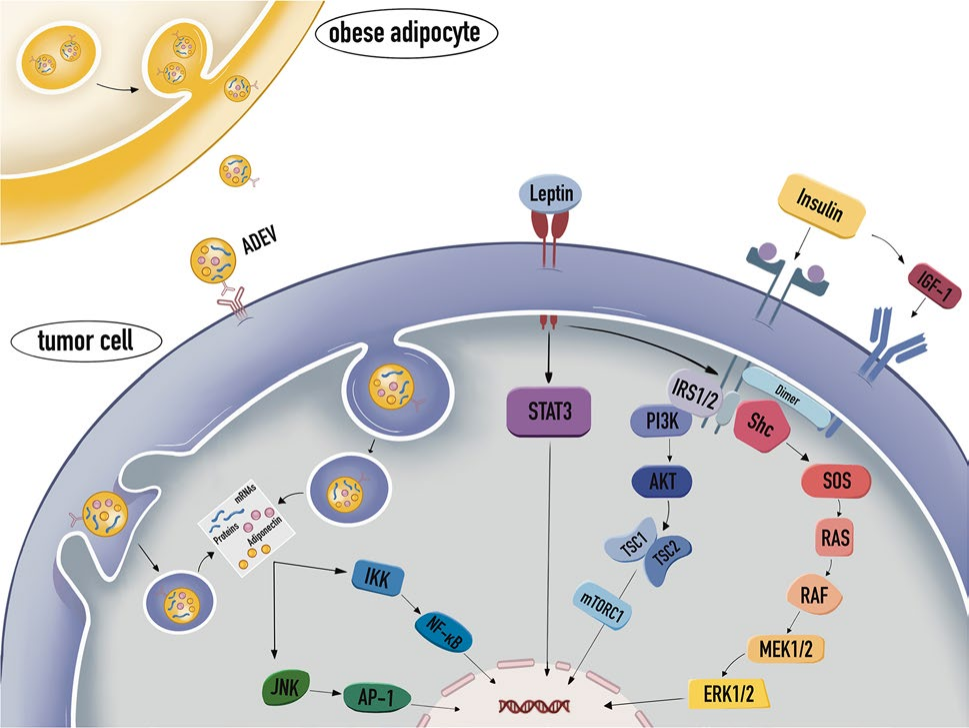
The pleiotropic roles of obese adipocyte secretome in cancer cell. In an obese state, adipocytes emit more leptin, increase the expression of IGF-1 in target cells, and release more ADEVs. Leptin increases P13K, STAT pathway signaling [48, 193]. IGF-1 is responsible for activating the RAS/RAF signaling network [194]. ADEV includes proteins, RNAs, and lipocalin that stimulate JNK/AP-1, IKK, and NF-KB signaling. These signaling mechanisms are triggered and promotes-the progression of cancer [34, 195]

Adipose Tissue (AT) is an endocrine organ capable of local and peripheral communication with other tissues as well as central communication with the appetite control region of the hypothalamus via the secretion of adipokines. It is also capable of communication with molecules involved in immune response and metabolic regulation via paracrine and/or endocrine action. When there is an imbalance between energy intake and expenditure, excess energy accumulates in adipocytes in the form of triglycerides, resulting in hypertrophy and hyperplasia. This leads to a rise in the bulk of adipose tissue, which becomes inflammatory and fibrotic. This myriad of events affects adipokine secretion, which contributes to the development of obesity-related problems [1] (Fig. 1).

### Obesity affects the nature, size, concentration of ADEV

In regards to obesity, a number of studies have been conducted via various techniques to characterize primary cell cultures of EVs from adipocytes or adipose tissue stem cell cultures, including in vitro cell cultures from mesenchymal cells differentiated into adipocytes. Also characterizing EVs isolated from whole adipose tissue secretions in different anatomical locations and in different metabolic states. According to a study by Connolly et al. [35], the adipocytes phases of development affect the contents in EVs or exosomes secreted by 3T3-L1 cells. High concentrations of adipogenesis indicators including peroxisome proliferator-activated receptor gamma (PPAR) and preadipocyte factor-1 (PREF1), as well as signaling fatty acids like arachidonic acid, can be found in these exosomes [35]. It’s interesting to note that, during the differentiation of 3T3-L1 cells, the level of adiponectin in the released exosomes peaks on day 15 [35]. In another study supporting these findings, ADEVs of mouse lineage released by C3H10T1/2 were characterized before and after differentiation, as well as after exposure to several therapies designed to enhance insulin resistance and adipose hypertrophy [36]. In order to understand cellular communication across tissues, including inflammatory signals and cellular interactions with the extracellular matrix (ECM), Camino et al. used a technique that retains the integrity of adipose tissue structure [37–39]. The findings of the research demonstrated that obese visceral adipocytes produce more EVs than subcutaneous adipocytes and had a greater protein concentration in EVs [36].

Different populations of EVs known as small extracellular vesicles (sEVs) and big extracellular vesicles (bEVs), are secreted by mature adipocytes [40]. In this research, Different sized ADEVs were found to have distinct proteome and lipidomic profiles, and their impacts on target cells were also found to vary, as determined by in vitro tests [40]. Another study reveals unique in vivo characteristics and functions of bEVs and sEVs in breast cancer, pointing to the significance of bEVs in illnesses and in applications for both diagnostic and therapeutic purposes [41]. The number of proteins found in bEVs and sEVs were 480 and 168, respectively. β-Actin, flotillin-2 and caveolin-1 are three unique peripheral proteins of bEVs that are involved in microvesicle shedding. Externalized phosphatidylserine is another attribute of bEVs. On the other hand, sEVs are enriched in tetraspanins CD9, CD63, CD81 Alix, and high cholesterol levels simultaneously [40, 41]. In addition, sEVs carrying enzymes involved in glycolysis and those carrying enzymes involved in fatty acid synthesis are secreted by white and brown adipocytes, respectively [42]. Compared to bEVs, which are characterized by substantial quantities of phosphoserine on the outer membrane leaflet, sEVs released by white adipocytes are significantly richer in cholesterol [40]. In a study by Wu et al. the surface proteins on these various ADEVs types have been profiled [43], which not only aids in identifying them but also offers possible clinical uses for these EVs [43, 44]. Nevertheless, The differences between the healthy and pathological roles of sEVs and bEVs generated by adipocytes are currently poorly understood.

Studies on the size, nature, and concentration of secretome released from AT have revealed higher concentrations of EVs in secretions from obese adipocytes depending on the site of origin and metabolic status (obese and lean). Thus indicating that the number and dynamics of secreted vesicle mechanics are highly dynamic and also regulated by the physiology of the cell of origin. We may thus assert that AT secretes vesicles of various sizes and qualities, the concentration of which is dependent on the tissue’s type and nutritional state.

### Obesity affects the cargo of ADEVs

Since the discovery of EVs, the idea that their cargo can act as disease markers has been incredibly intriguing. Due to the fact that the cargoes are exclusive to certain cell types, they can additionally serve as a marker of altered activity in a particular tissue, such as AT. Many illnesses have been shown to exhibit stage-dependent variations in cargo composition, highlighting the utility of EV cargo profiling for tracking disease development [45]. The function of EVs is largely governed by their cargo, to which membrane antigens and molecular carriers are crucial for their functional effects on target cells/tissues. Interestingly, the obesity implications on EV cargo sorting provide compelling examples of how EV biosynthesis and secretion might be targeted to control cell biology. Here, we focus on how obesity-related diseases affect cargo sorting in cell types.

**Fig. 2:**
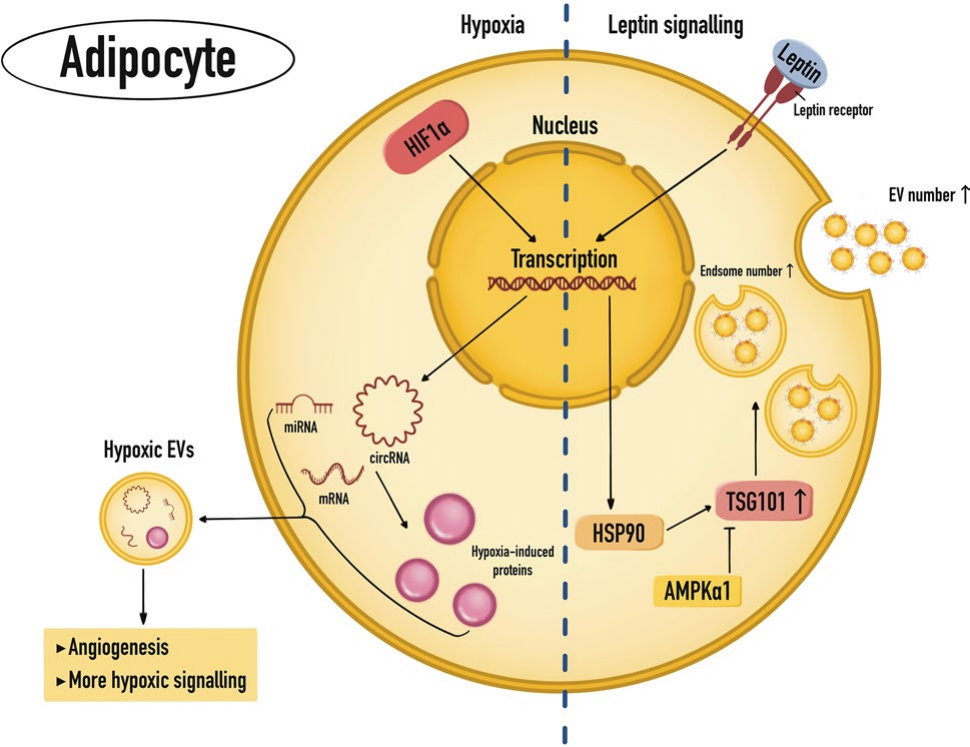
Obesity-leptin signaling and Hypoxia regulate cargo of the ADEV. Hypoxia activates HIF1α-mediated transcriptional upregulation of circRNAs, miRNAs, and mRNAs that, along with the proteins they encode, enter EVs [196]. Leptin signaling activates transcription of HSP90 [48, 50], which stabilizes ESCRT-I subunit TSG101 and increases endosomes and the number of EVs [50]. In contrast, AMPKα1 would negatively regulate TSG101 protein levels [197]

In obese individuals, ADEV size, number, and cargo composition are all changed, in part due to metabolic changes in the cellular concentrations of palmitic acid and ceramide [46–48]. There is a correlation between obesity and increased AT leptin secretion, with leptin enhancing the secretion of exosomes from breast cancer cell lines, which promotes oxidative metabolism and angiogenesis in the receiving cells [49]. Exosome production is stimulated by leptin via receptor-signaling HSP90, which in turn interacts with TSG101 to upregulate its protein expression [48, 50] (Fig. 2). The EVs have a higher concentration of HSP90 and other leptin signaling components, which further spreads the activation of leptin signaling in recipient cells [48]. Recent studies have also suggested substantial EV transfer across adipocytes, cardiac cells, -cells, macrophages, and cancer cells, indicating that EV dysregulation may underlie the development of metabolic comorbidities including obesity, cardiovascular disease, and cancer [18, 51].

Additionally, ADEVs may carry miRNA to influence the activity of recipient cells. miRNA functions as a post-transcriptional regulator of messenger RNA production and promotes changes in protein products [52]. An elegant study demonstrates that in genetically engineered mice lacking the miRNA-processing enzyme (Dicer) in the AT, circulating miRNA levels are drastically reduced, and this reduction can be reversed by transplanting a fat depot, indicating that adipose tissue is the primary source of circulating miRNA [53]. Under conditions of obesity, dysfunctional adipocytes will exhibit dysregulated exosome secretion, which contributes to alterations in circulating miRNA composition [54]. The ADEV-miRNA alterations associated with obesity have been summarized in Table 3. Changes in the content of circulating microRNAs may explain clinical conditions in obese people [55].

**Table 3:** The mechanism and function of miRNAs in the obese ADEVs

Source	Speice	Cargo	Target	Mechanism	Effect	Ref.
Isceral and subcutaneous adipose samples	Human	miR-23b miR-148b miR-4269 miR-4429	lung epithelial cells (A549)	Activating TGF-β and Wnt/β	Progression of chronic inflammation and fibrotic disease	[210]
Adipose tissue	Mice	miR-155	Macrophage	Activated STAT1 and suppressed STAT6	M1-M2 ATM polarization	[191]
		miR-223		Inhibition of translation of Pknox1		[211]
		miR-34a		Repressing Krüppel-like factor 4 (KLF4)		[212]
Adipose tissue	Mice	miR-34a	Hepatocyte	M1-M2 ATM polarization	Hepatic steatosis Glucose intolerance Insulin resistance	[212]
Adipose tissue	Mice	miR-141-3p	Hepatocytes	PTEN/AKT	Insulin resistance	[213, 214]
		miR-222		PPARγ/AKT		[215]
		miR-27a	Skeletal muscle			
PVAT	Mice	miR-221-3p	VSMCs	PGC-1α/PPARγ	Vascular remodeling	[216]
Adipose tissue	Mice	miR-23a/b	Hepatocytes	MALAT1/mTOR/POMC	Tumor proliferation	[217]

*KLF4* Krüppel-like factor 4, *MALAT1* metastasis associated lung adenocarcinoma transcript 1, *mTOR* mammalian target of rapamycin, *POMC* pro-opiomelanocortin, *PTEN* phosphatase and tensin homolog, *PPARγ* peroxisome proliferator-activated receptor γ, *PGC-1α* peroxisome proliferator-activated receptor gamma coactivator 1α, *VSMC* vascular smooth muscle cell, *PVAT* perivascular adipose tissue

### Hypoxia and EVs

A fall in tissue oxygen saturation is referred to as hypoxia. Obesity, lung illness, myocardial infarction (MI), and brain or limb ischemia are frequent pathological diseases linked to hypoxia [56–59]. Hypoxic TME is caused by an increase in oxygen demand or a rise in blood volume due to excessive cell proliferation [60, 61]. Hypoxia generates excess reactive oxygen species (ROS), causing oxidative stress, which then affects metabolic signaling and Hypoxic EVs (HypoEVs) content. The signaling pathway for hypoxia-inducible factor (HIF) is activated by hypoxia [62, 63]. Prolyl-4-hydroxylases (PHDs) quickly hydroxylate HIF-subunits in normoxic circumstances and route it to proteasomal breakdown. However, in the presence of hypoxia, this breakdown process is inhibited, and the HIF- subunits translocate into the nucleus where they attach to HIF-1 (HIF1B). The vascular growth factors VEGF-A and PDGF-B are among the more than 100 target genes that are upregulated as a result of the heterodimeric complex HIF binding to the hypoxia-responsive elements (HREs) within the promoter regions thus promoting tissue survival [64]. Additionally, hypoxia could activate HIF-independent signaling pathways, such as nuclear factor NF-kB [65], mTOR [66], and STAT3 pathways, hence boosting cell proliferation and inflammatory responses [67].

**Fig. 3:**
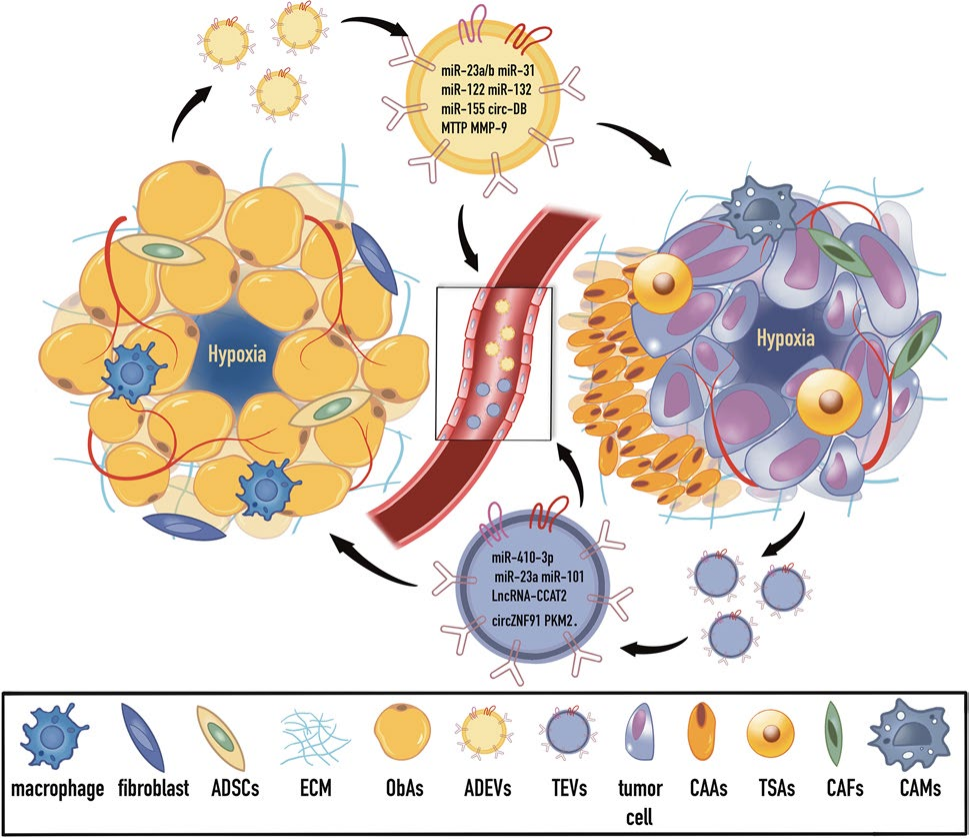
Extracellular vesicles: the cross-talk in obese ATME and TME. EVs play important roles in both obesity and TME. Obesity can lead to chronic inflammation, activate inflammatory cells in the TME, and the ADEV secreted by ObAs can induce and maintain the TME [198]. Meanwhile, tumor cells activate healthy AT by releasing EVs, leading to decreased lipid content and decreased adipocyte markers, which are referred to as CAAs [199]. CAAs are one of the main energy-supplying cells of tumor cells [161, 200]. In addition, EVs can also regulate metabolic pathways and signaling pathways of tumor cells in the pathological state of obesity, promoting tumor formation and progression. The special metabolic state of obesity also affects the composition and production mode of EVs, which reshapes TME and affects the death or survival of tumor cells while regulating metabolic networks. As an important signaling molecule or regulator, EVs play an important role in regulating tumor development. *ADSC* adipose-derived stem cells, *ECM* extracellular matrix, *ObAs* obese adipocytes, *ADEVs* adipocyte-derived extracellular vesicles, *TEVs* tumor cell-derived extracellular vesicles, *CAAs* cancer-associated adipocytes, *TSAs* tumor-stromal adipocytes, *CAFs* cancer-associated fibroblasts, *CAMs* cancer-associated macrophage

Cancer-related cells produce more EVs than healthy cells because intracellular communication or nutrient exchange is necessary [68] (Fig. 3). EVs are believed to be twice as common in the blood of cancer patients as in healthy individuals [69]. Therefore, it makes sense to assume that more exosomes are required to meet the needs of cell communication in cancer given the complex hypoxic environment that arises in tumors. The validity of this hypothesis has been demonstrated in several cancers, including glioma [70], breast cancer [71, 72], hepatocellular carcinoma [73], pancreatic cancer [74], gastric cancer [75], colorectal cancer (CRC) [76, 77], and prostate cancer [78], with various functions being mediated by exosome cargoes. Together, these findings show that hypoxia affects tumors by producing more cancer cell exosomes, which can communicate with neighboring cells. Interestingly, hypoxia also causes a rise in exosome production in non-cancerous cells [79, 80], demonstrating that exosome production is uniformly increased by hypoxia. However, it is still unclear how precisely hypoxia stimulates the release of exosomes from cancer cells.

### Hypoxia in obesity affects the cargo of the ADEVs

The main phases in EVs release are, cargo sorting, MVB transport, and fusion with the plasma membrane. Hypoxia may have an effect on all these steps. The recruitment of the membrane-anchored Ras superfamily of small G proteins (RABs) for membrane budding and fusion events is a recognized pathway for EV formation. There are hints that the activation of HIFs may immediately impact the RAB-dependent EV biogenesis pathways. The release of EVs from breast cancer cell lines was demonstrated to be induced by hypoxia, and this induction was abolished when HIF1 or 2 were silenced, indicating that the release of the EVs was HIF-dependent [81]. Additionally, it was discovered that HIFs directly attach to the RAB-A locus and trigger the production of RAB22A, a protein necessary for the budding of microvesicles from the plasma membrane. It was possible to stop the release of EV caused by hypoxia by inhibiting RAB22A expression. The observed enhanced release of EVs owing to hypoxia was found to be dependent on the direct binding of HIF1 to the RAB-A promoter in B cells [82]. Several tetraspanin membrane proteins, including CD81 and CD63, as well as TSG101, are exosome markers that are useful for detecting hypoxia regulation. Some of these markers are both sorting mediators and exosome cargo. Exosomes have a high concentration of CD63, which has been linked to its role in endosomal sorting during melanogenesis [82]. The sorting of diverse payloads into exosomes also directly involves the tetraspanins CD81, CD82, and CD9. Tetraspanin is shown by many researchers to be increased by hypoxia. For instance, poor outcomes for patients with GIST (gastrointestinal stromal tumors) are associated with overexpressed CD63 and GLUT-1 [83], which are markers of hypoxic state. These investigations inferred support for the hypothesis that cargo loading and EVs release may be impacted by exposure to hypoxia.

Furthermore, hypoxia modifies the nucleic acid composition of EVs. Under hypoxic circumstances, several miRNAs are transcriptionally activated by HIF1 and are hence more abundantly produced in EVs (Fig. 2). These include miR-210 and miR-21, which give cardioprotective anti-apoptotic effects in myocardial hypoxia models [84, 85]. In several cancer and cardiovascular disease models, HypoEVs include elevated concentrations of pro-angiogenic miRNAs [86–88]. Circular RNAs (circRNAs) may suppress these miRNA activities, and hypoxia signaling seems to fine-tune the downstream effects of EVs by modifying both the miRNA and circular RNA payloads. In hypoxic pancreatic cancer cells, for instance, the circular RNA circZNF91 is increased by HIF1 and delivered as EV cargo to recipient cells, where it functions as a sponge to suppress miR-23b-3p and facilitate additional HIF1-mediated transcriptional reprogramming [89]. The hypoxic increase of circRNA cargo in EVs is linked to ischemic heart disease, CRC, and diabetic retinopathy [90–93]. Hypoxia also induces HIF1-mediated overexpression of mRNAs (and their associated proteins) in glioma EVs, as well as altered levels of cardiac fibrosis-promoting lncRNAs in cardiomyocyte EVs [94, 95].

Hypoxia in adipocyte and AT is brought on by lipid overloading and the ensuing hypertrophy of adipocytes because of inadequate blood flow [96]. Meanwhile, the contents of EVs are similarly impacted by hypoxia. Adipose tissues experience hypoxia when the size of the fat pad rises. Hypoxia enhances the metabolic process-related adipocyte exosomal proteins [97]. A hypoxic state enhances the exosomal proteins involved in lipid syntheses, such as acetyl-CoA carboxylase, glucose-6-phosphate dehydrogenase, and fatty acid synthase, according to research using 3T3-L1 adipocyte models. In comparison to normoxic settings, these proteins are expressed at levels that are three to four times higher [97]. Obese patients have shown impacts of obesity on the exosomal cargo of adipocyte-derived exosomes. A clinical investigation demonstrates that obese patients’ subcutaneous adipocyte-derived exosomes are enriched in proteins associated with fatty acid oxidation [32]. This provides proof of a link between obesity and hypoxia, and the altering of EVs of adipocytes in a state of hypoxia.

Hypoxia is a key factor in AT dysfunction and an important pathophysiological phenomenon in obesity diseases and altered TME [98, 99]. Mature adipocytes, resident immune cells like macrophages, fibroblasts, and the stem cell population known as “preadipocytes” are all different cell types that make up the adipose tissue microenvironment (ATME) [100–102] (Fig. 3). The ATME is well-vascularized and abundant in anti-inflammatory cytokines (such as IL-4, IL-10, and IL-13) at healthy body weight settings (metabolic homeostasis), and as a result, harbors a variety of immune cells, including IL-4-producing eosinophils, group 2 innate lymphoid cells, M2 macrophages, and type 2T helper (Th2) cells. Adipocytes undergo hyperplasia and hypertrophy in response to body weight growth or metabolic obesity, as the vascular supply is constrained, these cells become stressed forming a hypoxic zone (100 μm away from functional blood vessels [103], with a partial pressure of oxygen < 10 mmHg [24]). This causes the release of damage-associated molecular patterns into the ATME, which causes innate immune cells (for example, dendritic cells, macrophages, and granulocytes) to invade and become activated [104], further participating in the chronic inflammatory response and altering the release of different EVs from adipocytes [105]. Moreover, elevated HIF-1 was detected in the AT of obese mice, and HIF-1 subunits have been directly linked to adiposity as well as the response to hypoxia [106, 107]. This is further supported by palmitate, a fatty acid that can harm cardiomyocytes by increasing ANT2 activity, which raises adipocyte oxygen consumption and HIF1 expression [108]. It is certain that HIF-1 expression is also enhanced in the AT of obese people [109, 110]. Curiously, HIF1 overexpression is both a marker of adipose tissue expansion and a contributor to that expansion, since it encourages further adipose growth by decreasing adipocyte fatty acid oxidation and raising intracellular fatty acid accumulation [111, 112]. Since adipocytes are crucial to the hypoxic microenvironment and TME, studying their cellular communication has been a popular area of study in recent years.

## ADEVs and hallmarks of cancer

The relationship between adipocytes and tumors has been better understood as a result of recent research on adipocyte-derived extracellular vesicles (ADEVs) with cancerous origin. By carrying fatty acids, adipose exosomes play a vital role in the development of cancer. They allow for metabolites, many non-coding RNAs, protein-degrading enzymes, and oxidative enzymes to enter cancer cells [11, 32, 113, 114]. Additionally, miRNAs, circRNAs, adipokines, and inflammatory components can be transported by tumor EVs to adipocytes, regulating AT development and substance release. This can cause the adipose to continue developing into CAAs, creating a setting for the survival and growth of cancer under hypoxic TME. Adipocytes-tumor cells crosstalk may therefore be mediated by EVs (Fig. 3).

**Fig. 4:**
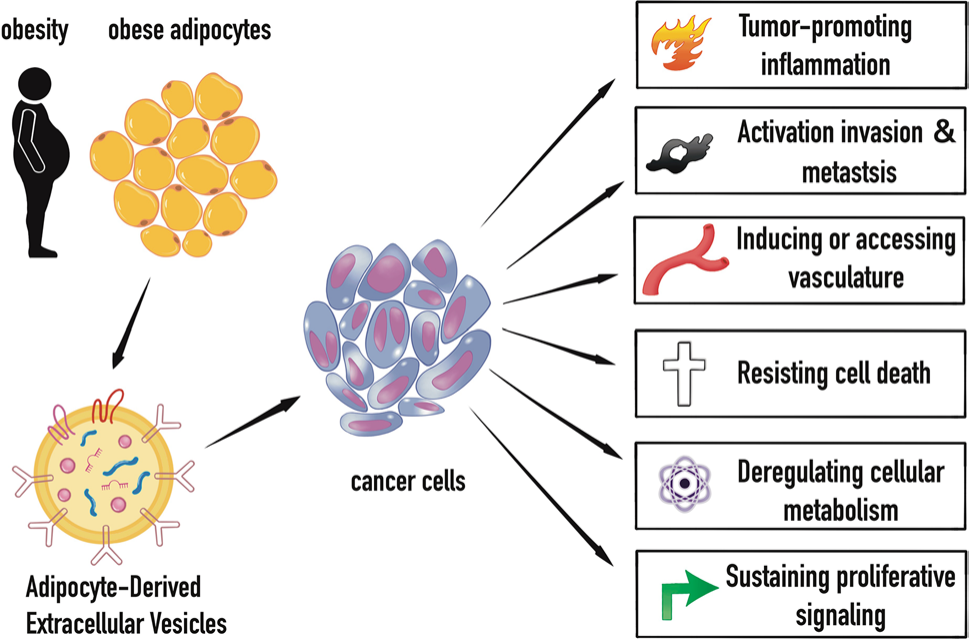
ADEV and the hallmarks of cancer under obesity. ADEV have been shown to play a crucial role linking obesity to the hallmarks of cancer, including but not limited to the tumor cell cycle, proliferation, migration, and metastasis, and thus promoting tumor formation and progression. ADEV can increase cancer cell proliferation through the activation of cAMP response element-binding (CREB) and extracellular signal-regulated kinase (ERK) signaling pathways [201], as well as induce tumorigenesis by regulating SOX-9 [202]. ADEV can stimulate cancer cell migration and metastasis by activating JAK/STAT-3 and TGF-β/SMAD signaling pathways [135]. Additionally, ADEV can regulate FAO and EMT processes to facilitate cancer cell metastasis by increasing the expression of matrix metalloproteinases (MMPs) and integrins to promote cell migration and invasion [32]. ADEV can also reduce apoptosis rates, promote malignant transformation, and advance malignancy progression cancer cells. Moreover, ADEV can alter cell transcriptomes and metabolomes, affecting cell survival and internal environment. Considering the crucial role of ADEVs in cancer cell growth, evolution, and metastasis, ADEV has become a new therapeutic target and an essential strategy for cancer treatment

Currently, few studies on EVs have concentrated on CAA EVs, with the majority of studies focusing on exosomes produced from cancer cells. In reality, tumor cells’ crosstalk with adipocytes results in evident structural and functional alterations to EVs [32]. For instance, proteins implicated in FAO are found in melanoma-associated adipocytes, which causes metabolic reprogramming in tumor cells. The combined impact of obesity and cancer, which worsens the symbiotic interaction between adipocytes and cancer cells, increases the number of exosomes released and their impact on tumor aggressiveness [32]. The capabilities that cancer cells have acquired were outlined by renowned cancer researchers Hanahan and Weinberg [8, 115], and as cancer has progressed, so has this knowledge. Literature shows, obesity, the microbiota, and autophagy are oncological factors [105, 116–118]. Numerous bio functions can be mediated by exosomes from cancerous or cancer-related cells, and hypoxia enhances these bio functions. Here, we give a few basic, illustrative, but not all-inclusive examples of several elements (Fig. 4; Table 4).

**Table 4:** Modified signatures and adipocyte-derived extracellular vesicles

Effect	Type of source	Content	Target	Mechanism	Ref.
Resisting cell death	ADMSC	N.D.	BCCs	N.D.	[218]
	ATM	miR-155	BCCs	Caspase-3, Bcl-2, APAR-1, FADD, and RIP1	[120]
	AT	miR-148b	BCCs		[122]
	ADMSC	N.D.	MCF-7	Wnt	[33]
	ADMSC	N.D.	MCF-7	TGF-β/Smad and P13K/AKT	[219]
	CAA	N.D.	Melanoma cells	Exchange of enzymes implicated in fatty acid oxidation	[11]
	AT	N.D.	MDA-MB-231 BCCs	PI3K/AKT	[34]
	AD	miR-23	HCC cells	VHL/HIF-1α axis	[114]
	AD	MTTP	CRC cells	MTTP/PARP/ZEB1	[123]
	ADMSC	miR-122	HCC cells	Cyclin G1, ADAM10, IGFR	[220]
Sustaining proliferation	AT	miR-144 miR-126 miR-155	CAAs (BCC)	Increased lipolysis in adipocytes tissue toward CAA phenotype	[128]
	AD	N.D.	Lung cancer cells	Increased MMP-3	[221]
	CAA	miR-21	Ovarian cancer cells	APAF-1	[222]
	AT	Circ-DB RNAs	HCCs	USP-7	[222]
	ADMSC	N.D.	Osteosaroma cells	COLGALT2	[127]
	AT	N.D.	BCCs	Increased CREB phosphorylation	[201]
	AD	miR-23	HCCs	Increased cell growth	[223]
	AT	N.D.	MCF-7 BCCs	ERK phosphorylation	[34]
Inducing angiogenesis	ADMSC	IL-8 CCL-2 VEGF-D	ECs	Increased migration and tube-like formation	[34]
	ADMSC	miR-132		Lymphangiogenesis via TGF-β/Smad signaling	[135]
	ADMSC	miR-31	HUVECs	Increased migration and tube-like formation	[137]
Activating invasion and metastasis	ADMSC	EGFR-1/IL-6	BCC	EGFR-1/IL-6 activating JAK/STAT-3 pathway	[141]
	AT	MMP-9	MDA-MB-231 BCCs	Increased invasive capacity	[34]
	AD	MMP-3	Lung cancer cells	Increased invasive capacity	[32]
	AD	N.D.	Melanoma cell Prostate cancer cell	Increase in melanoma cell migration and invasion; tumor progression in melanoma and prostate cancer by upregulating genes involved in fatty acid oxidation	[32]
Metabolism reprograming	AD	HCDH	Melanoma cell	Improving lipid metabolism, respiratory chain activity, and tumor migration	[32]
	AD	Mitochondrial FIS-1 and OPA-1	Melanoma cell	Induce mitochondrial redistribution to the edge of melanoma cells favoring migration	[11]
	AD	ECHA	Melanoma cell	Increased fatty acid oxidation	[11]
	AD	miR-433-3p	SCD1, nasopharyngeal carcinoma	promote lipid accumulation in NPC cells and facilitate proliferation and migration	[224]
Avoiding immune destruction and inflammation	ADMSC	N.D.	N.D.	Inactivation of T cells	[225]

*ADMSC* adipose tissue mesenchymal stem cells, *ATM* adipose tissue macrophages, *AD* adipocytes, *AT* adipose tissue, *BCC* breast cancer cells, *CAA* cancer-associated adipocytes, *EC* endothelial cell, *HCC* hepatocellular carcinoma, *HUVEC* human umbilical vein endothelial cells, *HCDH* hydroxyacylcoenzyme A dehydrogenase, *FIS-1* fission protein 1, *NPC* nasopharyngeal carcinoma

### Proliferation and resisting cell death

ADEVs have been shown to have an abnormally high concentration of miR-155, a microRNA that contributes to insulin resistance in obese people [119]. A proliferative/antiapoptotic impact of miR-155 in breast cancer cells is mediated via caspase-3, Fas-associated death domain (FADD), receptor-interacting protein 1 (RIP-1), and apoptosis peptidase activating factor-1 (APAF-1) [120]. Additionally, miR-155 targets the tumor protein P53 inducible nuclear protein 1 (TP531NP1) in MCF-7 breast cancer cells, providing resistance to cell death [121]. Via cell-to-cell contact, the miRNAs present in ADEVs can also operate as tumor suppressors by preserving homeostasis. For instance, miR-148b, which AT secretes into exosomes, suppresses tumor growth in breast cancer cells [122]. The significance of obesity-associated adipose tissue in malignant transformation is further supported by the fact that these miRNAs are downregulated and their pro-apoptotic activity is reduced in obese settings.

When the body is in an obese state, ADEVs can also transport micro proteins that impact ferroptosis in tumor cells [123]. Microsomal triglyceride transfer protein (MTTP) is abundantly expressed in AT and regulates lipid metabolism by promoting triglyceride transport between membrane vesicles [124], which is strongly associated with the prevention of ferroptosis. Proline-rich acidic protein 1 (PRAP1) colocalizes with MTTP in the endoplasmic reticulum (ER) and enhances MTTP-mediated lipid transport [125]. In a recent study on CRC, researchers discovered that elevated expression of MTTP inside ADEVs in individuals with a high BMI prevented lipid peroxidation through MTTP/PARP/Zinc finger E-box binding homeobox 1(ZEB1), which in turn inhibited ferroptosis [123]. These findings show that EVs released by adipose tissues actively contribute to cell homeostasis under normal circumstances. However, under obese circumstances, changes in their cargo composition may prevent tumor cells from dying off and speed up the progression of cancer.

ADSCs-derived EVs regulate proliferation of cancer cells through Wnt/β-Catenin signaling regulation [126]. The procollagen galactosyltransferase 2 (COLGALT2) pathway is activated by ADSC-derived exosomes, leading to increased proliferation and development of osteosarcoma cells [127]. Recent studies have demonstrated that breast cancer cells transport miRNA-rich exosomes to adipocytes, where they transform resident adipocytes into CAAs like miR-144, miR-12, and miR-155 [128]. Additionally, miR-122 promotes the development of the disease by reducing the glycolytic enzyme pyruvate kinase, which inhibits the uptake of glucose in premetastatic niche cells [128].

### Angiogenesis, invasion, and metastasis

Endothelial cells (ECs) migrate and multiply to form new blood vessels during angiogenesis, which is the process by which new blood vessels are formed from existing vascular networks [129]. Through the provision of additional nutrients and oxygen as well as the purification of waste materials, this mechanism supports tumor development [130]. An equilibrium between pro- and anti-angiogenic signaling pathways controls angiogenesis in physiological settings [131]. ECs migrate and multiply in metabolically difficult conditions, such as hypoxic and nutrient-deprived tissues, when this equilibrium is broken in tumor tissues. The stromal vascular fractions of adipose tissue produce a high number of proteins involved in angiogenesis, wound healing, and tissue regeneration [131, 132]. The secretome of adipose tissue also contains pro-angiogenic proteins. Recent research has examined the involvement of EVs in hypoxia-induced tumor angiogenesis in an increasing number of investigations [133].

For instance, hypoxic extracellular vesicles (HypoEVs) produced from extremely malignant glioblastoma multiforme cells promote pericyte migration and angiogenesis by encouraging EC release of cytokines and growth factors in adipose tissue [94]. Moreover, the study showed that angiogenic proteins such as VEGF-D, CCL2, and IL-8 were absorbed by ECs in the EVs generated by ADMSCs. The ECs undergo differentiation after internalization, produce more migration, go through a tube-like structure in a test tube, and encourage angiogenesis in a living organism [134]. In a study by Wang et al. study, ADMSCs treated with VEGF-C release EVs enriched in miR-132, demonstrating another use for ADMSCs-derived EVs [135]. Through controlling TGF-/Smad signaling, miR-132 translocation to lymphatic ECs encourages lymphangiogenesis, a process that takes place during tumor spread [136]. AT from obese patients has increased levels of miR-31, which is linked to angiogenesis, compared to healthy participants [137]. Human umbilical vein endothelial cells (HUVECs) can migrate and form tubes when exposed to exosomes made from ADMSCs, as shown by Kang et al. [138].

Cancer cells can migrate from the main tumor by an epithelial-to-mesenchymal transition (EMT), and then colonize a secondary site through a mesenchymal-to-epithelial transition during metastasis (MET). At both the primary and secondary tumor locations, each of these occurrences modifies the TME. Via EMT, invasion is induced by the transformation of tumor cells, which is accompanied by the production of a pro-inflammatory tumor-associated stroma. Intrinsic factors, such as the activation of signaling pathways, transcription factors, microRNAs, or epigenetic modifications, control the onset of EMT. These intrinsic factors are in turn impacted by extrinsic factors, such as interactions between the tumor and the stroma [139, 140]. The Janus kinase (JAK)/signal transducers and activators of the transcription (STAT-3) pathway are activated by the epidermal growth factor receptor 1 (EGFR-1) and IL-6 in patients with type 2 diabetes mellitus, which results in the migration and metastasis of breast cancer cells [141]. Furthermore, these vesicles induced the upregulation of genes involved in breast cancer cell migration, such as C-X-C chemokine receptor type 4 (CXCR4) and vascular endothelial growth factor C. These vesicles also upregulated genes involved in metastasis, including tumor growth factor (TGF-b), basic fibroblast growth factor (bFGF), and epidermal growth factor (EGF). According to this theory, Ramos-Andrade et al. have previously shown that the transfer of MMP-9 from EVs produced from obese adipose tissue increases the invasive potential of MDA-MB-231 cells [34]. Studies of HypoEVs from CRC [142], bladder cancer [143], gastric cancer [75], and lung cancer have demonstrated the role of HypoEVs in driving invasion and metastasis.

### Immune response

Bioactive compounds, especially chemicals and reactive oxygen species (ROS), may be introduced into the TME as a result of inflammation. These molecules are highly mutagenic to the surrounding cancer cells, hence promoting their genetic development toward greater malignancy. Given that inflammation aids in the formation of these essential signature abilities, it is seen as a facilitating trait [8]. Obesity is associated with an increased risk for developing cancer and a poor prognosis, as shown by the release of ADEVs in the TME.

Sun et al. demonstrated that AT is directly involved in chronic inflammation inside the breast cancer microenvironment, where it promotes adipocyte hyperplasia and cytokine-related signaling pathways in macrophage. Additionally, they demonstrated that the formation of breast cancers is driven by macrophage infiltration in AT [144]. In a similar vein, Nieman et al. demonstrated that adipocytes in the omentum secrete adipokines like IL-8, which promote the invasion and migration of ovarian carcinoma cells [145]. Zhao et al. found that when macrophages took up mouse exosomes produced by ADMSCs, mRNA levels of arginase-1 and interleukin-10 increased while mediating phosphorylated STAT-3, leading to polarization of macrophages toward the M2 phenotype [146]. It’s crucial to remember that Tumor-associated macrophages (TAMs) behave similarly to M2 macrophages in the tumor microenvironment [147]. M2-macrophages, which lack activity and phagocytic capacities, generate and release growth factors (fibroblast growth factor [FGF], macrophage-colony stimulating factor [M-CSF], and platelet-derived growth factor [PDGF]). Tumor growth is promoted by the immunosuppressive nature of the microenvironment and the growth factors PDGF, TGF-1, and VEGF [148].

Since the success of checkpoint blockade (such as with drugs that target PD-1 or PD-L1), immunotherapy has established itself as a viable cancer therapeutic option. Programmed cell death ligand 1 (PD-L1) overexpression inhibits the anticancer effects of cytotoxic T lymphocytes by causing inhibitory signaling [149]. A decrease in CD8+ and fatigue of tumor-infiltrating lymphocytes have been linked to increased tumoral expression of PD-L1 in hepatocarcinoma and melanoma in an obesity-related animal model. Intriguingly, they demonstrated that TNF- and IL-6 generated by adipocytes still had a comparable effect even after both cytokines had neutralized the expression of PD-L1 [150]. The first immune response regulator in terms of signaling suppression is the surface expression of ligands or receptors, and HypoEvs can alter this expression. Oral squamous cell carcinoma (OSCC)-derived HypoEVs miR-21 promotes the expression of PD-L1 in ADSCs, which reduces the antitumor activity of T cells [151].

### Metabolism reprogramming

Metabolic reprogramming, such as the Warburg effect, occurs in cancer cells even under normoxia [152]. Investigating the involvement of EVs in metabolism in the hypoxic TME is crucial since metabolism has significant effects on cancer biology. Glycolysis enhances tumor cell metabolism by facilitating the dysregulation of cellular energy complexes such as the production of biomolecules through the pentose phosphate route rather than from intermediary molecules. Once the oxygen in the tumor microenvironment is depleted, transcription factors like HIF-1 are activated, which encourages the action of the glycolytic enzymes [8, 115].

The breast, subcutaneous, periprostatic, and visceral abdominal fat found in appendices epiploic ae, mesentery, and omentum are among the most adipose-rich tissues in humans. The “success” of tumor formation in tumors growing next to these adipose depots depends on the tumor’s capacity to trigger metabolic change and rewire adipocytes to support tumor growth [153, 154]. The adipocytes’ energy reserves of lipids and long-chain fatty acids (FA) are accessed by tumor cells and used as their main energy source. When the demand for adenosine triphosphate (ATP) is high during rapid tumor growth, loss of attachment to the extracellular matrix, and metastasis, fatty acid oxidation (FAO) becomes essential for cancer cells [155]. Fatty acids may enter a tumor through interactions with adipocytes in the TME or through the bloodstream. Following cellular absorption, FA are retained in tumor cells as lipid droplets, which are then released during lipolysis. Monoacylglycerol lipase (MAGL), an enzyme that releases free fatty acids (FA) from monoacylglycerol during lipolysis, is found in abundance in aggressive cancer cell lines. Tumor growth and cancer cells’ migration capacity are both decreased when MAGL is inhibited [156]. Breast, prostate, and melanoma cancers interact metabolically with CAAs locally, switching the cancer cells’ metabolic pathway from glycolysis to lipid-dependent energy production. Ovarian, colon, and stomach tumors also engage in this metabolic symbiosis.

ADEVs have been shown to interact with melanoma, breast, and ovarian cancer cells in recent investigations, regulating their metabolism and boosting several malignant features [157, 158]. Examples of fatty acid metabolism-related enzymes found in ADEVs include trifunctional enzymes and 3-hydroxyacyl-CoA dehydrogenase. They boost melanoma cell motility, lipid metabolism, and respiratory chain activity through affecting the fatty acid oxidation cycle in malignant cells [32]. Cancer cells interact with adipocytes, which provide the TME with FFA, leptin, ketone bodies, and other macromolecules that alter cancer cell metabolism. The Warburg effect may also be triggered when cancer cells acquire glycolytic enzymes from adipocytes in the bone marrow. After being taken in, cells undergo oxidative stress, releasing ROS into the TME, which triggers the “reverse Warburg effect” and initiates aerobic glycolysis, leading to the production of high-energy metabolites [127]. Furthermore, EVs produced by cancer cells carry glycosidases, which can break down ECM constituents like glycoproteins and proteoglycans within the ECM thereby remodeling it and encouraging tumor development [159]. According to a metabolomics investigation, MSCs-derived EVs contained glutamate and lactate. Lactate may help cancer cells survive in hypoxic and nutrient-poor conditions, whereas glutamate may provide precursors for the major macromolecular classes through the movement of carbon and nitrogen [160]. Additionally, carbohydrates and amino acids are transported between cancer cells and CAAs in EVs, providing a steady supply of fuel and building blocks for the tissue [128]. Wu et al. found serval miRNAs in EVs, such as miR-105, miR-122, miR-126, and miR-155, are essential for reprogramming the energy metabolism in CAA and breast cancer cells [128].

## Obesity to cancer progression and metastasis

Growing evidence suggests the link between obesity and cancer [157–159]. Obesity may not only contribute to carcinogenesis, but also play an important role in tumor development and metastasis [101]. EVs consist of plasma-transported vesicles released by human tissues and indicative of metabolic state. In metabolic illness, the profile of exosomes (especially their microRNA content) is changed [160]. EV circulating in type 2 diabetes mellitus (T2DM) plasma promote transcriptional alterations associated with tumor progression and pro-metastatic characteristics in target cancer cells, thereby connecting obesity to cancer progression and metastasis [161].

### Obesity and tumorigenesis

Recent research has emphasized the contributions of the triad of overweight/obesity, insulin resistance (IR), and adipocytokines to cancer. Although the role of obesity in cancer etiopathogenesis is still not completely remains incompletely understood, the primary pathways connecting obesity and cancer include: (1) hyperinsulinemia/IR and abnormalities in the IGF-1 system and signaling; (2) sex hormone biosynthesis and pathways; (3) subclinical chronic low-grade inflammation and oxidative stress; (4) alterations in adipocytokine pathophysiology; (5) factors originating from ectopic fat deposition; (6) microenvironmental and cellular perturbations; (7) circadian rhythm disruption and dietary nutrients; (8) altered gut microbiome; (9) mechanical factors in obesity; (10) extracellular matrix remodeling and angiogenesis; (11) adrenergic signaling. Figure 1 illustrates the mechanisms linking obesity to cancer.

### Obesity and tumor progression

Chronic inflammation brought about by obesity has the potential to not only cause tumors to form, but also to supply tumor cells with growth factors and inflammatory factors in the microenvironment of the tumor that are helpful to the advancement of the cancer [7, 101, 162]. These under this conditions, triggers alterations of normal leptin and adiponectin levels, which in combination with the co-occurrence of other changes, including infiltration of macrophages, mitochondrial dysfunction and increased ER stress response may be associated with promotion of cancers such as CRC in obese individuals [163–165]. Because they are a source of energy, High quantities of FFAs seen in obese AT may encourage the formation of tumors due to them being hey are a sources of energy [166]. In addition, obesity may increase the resistance of tumor cells to apoptosis, reflecting resistance to cell death and sustained proliferation as hallmarks of cancer [167].

### Obesity and tumor metastasis

The composition and architecture of the cellular matrix can be altered by obesity, which can make it easier for tumor cells to invade and migrate through the body [123]. According to a number of studies, obesity can encourage tumor cells to create a greater ability for migration, which in turn promotes the spread of tumor metastasis [168]. In BC, adipocytes of tumor-stromal interface CAAs acquire a fibroblast-like phenotype linked with increased invasiveness via the production of different proteases and cytokines [169]. It is possible that the ADEV seen in the AT of obese people have a role in the promotion of tumor metastasis. This involvement may involve modifying TME as well as encouraging the migration of tumor cells.

### ADEV bridging obese ATME and TME

ATME and TME are frequently linked and interact, and the transport of ADEV is a key means of information transmission [18]. The results of previous research indicate that ADEV not only reflects the metabolic condition of obese individuals, but also provides functional instructions that may differentially induce tumor growth [32, 161, 162]. This may explain why cancer patients with obesity and/or metabolic illness have more advanced tumors and lower outcomes than those with normal metabolism (these include shorter disease-free survival and greater risk of recurrence of obesity-related cancers). The ATME in BC and obese individuals with metabolic problems, as well as the accompanying inflammation, may interact with malignant cell clones via cytokines, chemokines, metabolites, and ADEVs [96, 162]. We propose that this TME is associated with more transcriptional plasticity and metastatic behavior than the ATME of individuals with a normal metabolism.

Milbank et al. suggested that specific modulation of hypothalamic AMPK using a sEV-based technology may be a suitable strategy against genetic forms of obesity [163]. A recent study showed that EV-induced proliferation and mitochondrial activity are associated with stimulation of the Akt/mTOR/P70S6K pathway, and are reversed upon silencing of P70S6K. This study reveals a new facet of the obesity-breast cancer link with human breast ADEVs causing the metabolic reprogramming of ER+ breast cancer cells [164]. ADEVs deliver insulinotropic cargo to pancreatic-cells. ADEV proteins were subjected to phosphorylation upon transfer, which boosted insulinotropic GPCR/cAMP/PKA signaling by boosting total protein abundances and phosphosite dynamics, and eventually increased glucose-stimulated first-phase insulin secretion (GSIS) [165]. In conclusion, drug delivery is a promising use of ADEV; for instance, novel engineering and development methods are being created for therapeutic targeting. The subject of ADEV biology is advancing quickly, however the ideal application of exosomes in precision medicine has not yet been determined.

## Clinical applications of ADEV

Further research might evaluate how obesity influences the interaction between ADEVs and target cells, as well as investigate the physiological and pathological functions of these ADEVs. ADEVs also play a significant role in how obesity affects TME, particularly hypoxia TME, by serving as “signaling station” or “processing plant” in cellular communication. Therefore, Hypoxic ADEVs in physiological fluids may be employed as a surrogate biomarker of hypoxia and for cancer prediction.

### EV as biomarkers for diagnosis of cancers

The fields of precision oncology and liquid biopsy are making strides forward with the expansion of precision medicine. Tissue biopsies often only sample from one or a small number of locations, which may not be representative of the tumor’s spread across the body. Liquid biopsy procedures, which include the analysis of circulating tumor DNA (ctDNA), EV, CTCs, and other biochemicals, seem to be able to get over these limitations [166, 167]. EVs are advantageous indicators for monitoring dynamic intratumoral heterogeneity because of their various biochemical components, which comprise not only DNA but also a range of proteins, RNA, glycoconjugates, and lipids, indicating further potential therapeutic utility.

EVs isolated from preoperative plasma samples of 40 HCC patients revealed that those with higher levels of exosomal miR-155 were significantly more advanced than those with lower levels [73]. The Kaplan–Meier analysis of survival revealed that disease-free survival was significantly lower in the miR-155 high group compared to the miR-155 low group. In addition, thirty surgically removed HCC specimens indicated a correlation between HIF-1 expression and exosomal miR-155. This study also indicated that under hypoxic conditions, HCC cell cytoplasmic and exosomal miR-155 expression was greatly increased [73]. The use of extracellular miRNAs as indicators of sickness is very attractive because of the specificity and sensitivity of miRNA detection [168–170]. Several circulating microRNAs, including miR-15a, miR-22, miR-92a, miR-122, and miR-192, have been shown to have a favorable correlation with obesity. However, findings might differ based on the isolation technique of the circulating miRNAs. To date, whole blood, serum, or plasma have been employed in the vast majority of studies examining circulating miRNAs. Many researchers have used macromolecular crowding reagents like polyethylene glycol or differential ultracentrifugation to separate sEVs from serum or plasma. The precision and repeatability required to consider a molecule as a biomarker may be considerably impacted by these various methods. The use of circulating miRNAs as reliable biological markers of health and sickness will be facilitated by the widespread adoption of standardized methodologies and procedures for the separation of circulating miRNAs, in combination with technologies that can identify their tissue of origin.

Compared to source cells, EVs are often abundant in sphingomyelin, phosphatidylserine, phosphatidic acid, ceramide, and cholesterol [171]. EV lipids are important for tumors to look the way they're supposed to, but they may also serve as signal molecules in many biological processes [171]. Using high-throughput mass spectrometry and quantitative lipidomics, Skotland et al. confirmed the diagnostic value of exosomal lipids in prostate cancers and identified multiple lipids in exosomes isolated from the urine and cell culture supernatants of patients with prostate cancer and healthy controls [172]. Similarly, patients with prostate cancer had their urine, platelets, and exosomes examined before and after prostatectomy using targeted ultra-high performance liquid chromatography with tandem mass spectrometry. The findings demonstrated that the small-molecule metabolites in exosomes were linked to cancer [173]. In addition, EVs in feces have potential as a biomarker for the early diagnosis of CRC [174]. This conclusion is supported by the discovery of two transmembrane proteins, CD147 and A33, on feces-derived extracellular vesicles (fEVs) that are inherently related with colorectal cancer. The detection findings reveal that the levels of CD147 and A33 on fEVs were significantly elevated in CRC patients, therefore separating them from healthy donors in a striking manner.

The prospect of integrating exosomal protein, lipid, RNA, and miRNA in cancer diagnosis and prognostic assessment is now being studied, given the current sophistication of analytical techniques. Using a mix of exosomal biomarkers, such as metabolites, RNAs, and proteins that uniquely represent disease characteristics, may increase the specificity and sensitivity of exosome-based cancer detection [69]. Cho et al. established a technique for multiplexed in situ detection of exosomal miRNAs and proteins that enabled the quantitative investigation of many disease-specific miRNAs and surface proteins in prostate cancer cell-derived exosomes in a single procedure [175].

### EVs as drugs or carriers for antitumor therapy

For the majority of medications, just a tiny quantity reaches the lesion to have a therapeutic impact. This decreases the effectiveness and might cause toxicity and significant side effects to the patient. EVs, as natural carriers of intercellular information, play a role in the interchange of biomolecules between cells; hence, they offer tremendous promise as innovative drug carriers. EVs offer several benefits, including their tiny size, inherent molecular transport characteristics, and high biocompatibility [176]. Meanwhile, exploring the processes through which adipocytes or EVs generated from AT influence obesity and cancer may aid in the development of novel treatment options. In a mice model in which deletion of adipocyte-specific Sirt1(which results in obesity) was done [177], therapy with an exosome production inhibitor, GW4869, dramatically decreased body weight, enhanced insulin sensitivity, and inhibited carcinogenesis [178].

Researchers found that two subtypes (CD90 high and CD90 low) of ADSCs have different anti-tumor activities [179]. CD90-low ADSCs and their EVs significantly inhibited tumor growth in a mouse breast cancer model. This inhibition was associated with reduced tumor cell proliferation and migration and enhanced apoptosis of tumor cells mediated by ADSC-EVs. This work attempts to utilize recently found anticancer ADSCs and ADSC-EVs in the clinical treatment of breast cancer and offers evidence that EVs may be employed as a novel and effective therapeutic strategy or drug delivery vesicles [179].

Additionally, ADSCs may be genetically engineered to carry tumoricidal genes or interferons against neoplastic cells, and this strategy has shown promise in mouse models of lung cancer, gliomas, Kaposi’s sarcoma, and melanoma [180]. Curcumin is a natural polyphenol molecule that has been found to have anti-inflammatory effects in a number of studies [181]. EVs might carry paclitaxel, doxorubicin, and temozolomide [182, 183]. Therapeutic exosomes might potentially transport small interfering RNAs (siRNAs) or anti-miRNA oligonucleotides [184]. Furthermore, cutting-edge technologies such as nanoparticles (cationic liposomes) carrying tumor RNAs, dubbed RNA lipoplexes (RNA-LPX), have been created [185–187]. This class of RNA-LPXs was shown to activate the immune system. Intriguingly, MSCs have the remarkable characteristic of migrating and localizing in inflammatory and damaged microenvironments, such as solid tumors [188]. As a result of this characteristic, MSCs may promote tumor growth while also inhibiting tumor growth, and MSC-derived EVs have been proposed as a possible anticancer vaccination or medication delivery approach [189, 190].

To summarize the above, ADEVs have the advantages of lipid affinity and engineering feasibility, which make them a promising candidate for future tumor diagnosis and treatment.

## Perspective

As ADEV research continues to deepen, it has been shown to be widely present in the body and has broad potential applications in biology and medicine [18]. Future research on ADEVs may concentrate more on therapies for obesity-related comorbidities, the development of biomarkers to aid in the early detection of malignancies, and the creation of cancer-specific medications.

1. Interventions for obesity and obesity-related diseases: the cargo of ADEVs in the obese state can be taken up by different types of cells, thus inducing a number of different pathological conditions. Notably, specific ligand/receptor interactions have been reported to mediate the uptake of ADEVs by T cells and to receive the effects of obesity. Blocking or altering ADEV uptake may have a great therapeutic potential. Obesity brings about chronic inflammatory and hypoxic niche in ATME, and studies based on the joint action of these two microenvironments will gradually receive attention. In particular, EVs of macrophages and ADSC in Obese-ATME have been studied, acting on insulin resistance and angiogenesis [191, 192].
2. ADEV-loaded gene editing technology: new information regarding the molecular connection between ADEVs and cancer cells may help the development and execution of innovative, long-term therapeutics employing novel chemicals or repurposing existing medications established for other disease situations that target particular pathways in these cells. These medications might be utilized to directly target adipocytes and/or cancer cells as stand-alone therapies or as adjuvant medicines to maximize the efficacy of existing treatments. By using gene editing technology to load RNA or proteins onto EVs and then releasing them into recipient cells, gene expression patterns can be manipulated or altered. This technology is being studied for its potential in treating various diseases.

Despite their numerous benefits, ADEVs present significant challenges in clinical applications, such as limited targeting efficacy and vulnerability to immune cell phagocytosis. Additionally, the procedure for isolating and purifying ADEVs is time-consuming and labor-intensive. ADEVs demonstrate considerable variability as well. The content of exosomes changes based on the kind of cells that make them, a factor that must be regulated in a therapeutic environment.

## Conclusion

In this review, we provide evidence supporting the crucial function of ADEVs in obesity and TME, as well as future potential as a therapeutic agent and biomarker for cancer. Despite the fact that complete comprehension has not been attained, the following is a summary:

Obesity increases the risk of malignancy and affects the prognosis of malignancies by altering receptor cells via ADEVs. Recent research has highlighted the role of ADEVs in the development and progression of numerous forms of cancer. This kind of communication may take place both practically and systematically. A hypoxic TME can be produced by obese ATME. To survive in a hypoxic microenvironment, tumor cells a variety of responses including producing ADEVs that transfer signals to other cells inducing cancer-promoting or protective effects. Hypoxic TME in obese AT produces exosomes that transfer signals to induce cancer-promoting or protective effects. Different EV loads exist in hypoxic and normoxic TMEs. Hypoxic TME lead to a greater quantity of vesicle released. In contrast, EVs from healthy adipose tissue contain an abundance of tumor suppressor molecules. ADEVs control cancer hallmarks by directing various cancer cell functions. Blocking AT expansion and inflammation, and understanding these processes, may offer possible solutions.

## Data Availability

No data was used for the research described in the article.

## References

[1] Balistreri CR, Caruso C, Candore G (2010). The role of adipose tissue and adipokines in obesity-related inflammatory diseases. Mediat Inflamm.

[2] Lobstein T, Jackson-Leach R, Powis J, Brinsden H, Gray M. World obesity atlas 2023 report. https://www.worldobesityday.org/World_Obesity_Atlas_2023_Report.

[3] Renehan AG, Tyson M, Egger M, Heller RF, Zwahlen M (2008). Body-mass index and incidence of cancer: a systematic review and meta-analysis of prospective observational studies. Lancet.

[4] Bhaskaran K, Douglas I, Forbes H, dos-Santos-Silva I, Leon DA, Smeeth L (2014). Body-mass index and risk of 22 specific cancers: a population-based cohort study of 5.24 million UK adults. Lancet.

[5] Forouzanfar MH, Afshin A, Alexander LT, Anderson HR, Bhutta ZA, Biryukov S, Brauer M, Burnett R, Cercy K, Charlson FJ, Cohen AJ (2016). Global, regional, and national comparative risk assessment of 79 behavioural, environmental and occupational, and metabolic risks or clusters of risks, 1990–2015: a systematic analysis for the global burden of disease study 2015. Lancet.

[6] Goncalves MD, Lu C, Tutnauer J, Hartman TE, Hwang SK, Murphy CJ, Pauli C, Morris R, Taylor S, Bosch K, Yang S, Wang Y, Van Riper J, Lekaye HC, Roper J, Kim Y, Chen Q, Gross SS, Rhee KY, Cantley LC, Yun J (2019). High-fructose corn syrup enhances intestinal tumor growth in mice. Science.

[7] Rathmell JC (2021). Obesity, immunity, and cancer. N Engl J Med.

[8] Hanahan D, Weinberg RA (2011). Hallmarks of cancer: the next generation. Cell.

[9] Schito L, Semenza GL (2016). Hypoxia-inducible factors: master regulators of cancer progression, trends. Cancer.

[10] Semenza GL (1863). The hypoxic tumor microenvironment: a driving force for breast cancer progression. Biochim Biophys Acta.

[11] Clement E, Lazar I, Attané C, Carrié L, Dauvillier S, Ducoux-Petit M, Esteve D, Menneteau T, Moutahir M, Le Gonidec S, Dalle S, Valet P, Burlet-Schiltz O, Muller C, Nieto L (2020). Adipocyte extracellular vesicles carry enzymes and fatty acids that stimulate mitochondrial metabolism and remodeling in tumor cells. EMBO J.

[12] Rosen ED, Spiegelman BM (2014). What we talk about when we talk about fat. Cell.

[13] Bartelt A, Heeren J (2014). Adipose tissue browning and metabolic health. Nat Rev Endocrinol.

[14] Bussard KM, Mutkus L, Stumpf K, Gomez-Manzano C, Marini FC (2016). Tumor-associated stromal cells as key contributors to the tumor microenvironment. Breast Cancer Res.

[15] Wang YY, Attané C, Milhas D, Dirat B, Dauvillier S, Guerard A, Gilhodes J, Lazar I, Alet N, Laurent V, Le Gonidec S, Biard D, Hervé C, Bost F, Ren GS, Bono F, Escourrou G, Prentki M, Nieto L, Valet P, Muller C (2017). Mammary adipocytes stimulate breast cancer invasion through metabolic remodeling of tumor cells. JCI Insight.

[16] Attané C, Milhas D, Hoy AJ, Muller C (2020). Metabolic remodeling induced by adipocytes: a new Achilles’ heel in invasive breast cancer. Curr Med Chem.

[17] Hartwig S, De Filippo E, Göddeke S, Knebel B, Kotzka J, Al-Hasani H, Roden M, Lehr S, Sell H (2019). Exosomal proteins constitute an essential part of the human adipose tissue secretome. Biochim Biophys Acta Proteins Proteom.

[18] Rome S, Blandin A, Le Lay S (2021). Adipocyte-derived extracellular vesicles: state of the art. Int J Mol Sci.

[19] Colombo M, Raposo G, Théry C (2014). Biogenesis, secretion, and intercellular interactions of exosomes and other extracellular vesicles. Annu Rev Cell Dev Biol.

[20] Salido-Guadarrama I, Romero-Cordoba S, Peralta-Zaragoza O, Hidalgo-Miranda A, Rodríguez-Dorantes M (2014). MicroRNAs transported by exosomes in body fluids as mediators of intercellular communication in cancer. Onco Targets Ther.

[21] Azmi AS, Bao B, Sarkar FH (2013). Exosomes in cancer development, metastasis, and drug resistance: a comprehensive review. Cancer Metastasis Rev.

[22] Zhang HG, Grizzle WE (2014). Exosomes: a novel pathway of local and distant intercellular communication that facilitates the growth and metastasis of neoplastic lesions. Am J Pathol.

[23] Huang T, Song C, Zheng L, Xia L, Li Y, Zhou Y (2019). The roles of extracellular vesicles in gastric cancer development, microenvironment, anti-cancer drug resistance, and therapy. Mol Cancer.

[24] Théry C, Witwer KW, Aikawa E, Alcaraz MJ, Anderson JD, Andriantsitohaina R, Antoniou A, Arab T, Archer F, Atkin-Smith GK, Ayre DC, Bach JM, Bachurski D, Baharvand H, Balaj L, Baldacchino S, Bauer NN, Baxter AA, Bebawy M, Beckham C, BedinaZavec A, Benmoussa A, Berardi AC, Bergese P, Bielska E, Blenkiron C, Bobis-Wozowicz S, Boilard E, Boireau W, Bongiovanni A, Borràs FE, Bosch S, Boulanger CM, Breakefield X, Breglio AM, Brennan MÁ, Brigstock DR, Brisson A, Broekman ML, Bromberg JF, Bryl-Górecka P, Buch S, Buck AH, Burger D, Busatto S, Buschmann D, Bussolati B, Buzás EI, Byrd JB, Camussi G, Carter DR, Caruso S, Chamley LW, Chang YT, Chen C, Chen S, Cheng L, Chin AR, Clayton A, Clerici SP, Cocks A, Cocucci E, Coffey RJ, Cordeiro-da-Silva A, Couch Y, Coumans FA, Coyle B, Crescitelli R, Criado MF, D'Souza-Schorey C, Das S, Datta Chaudhuri A, de Candia P, De Santana EF, De Wever O, Del Portillo HA, Demaret T, Deville S, Devitt A, Dhondt B, Di Vizio D, Dieterich LC, Dolo V, Dominguez Rubio AP, Dominici M, Dourado MR, Driedonks TA, Duarte FV, Duncan HM, Eichenberger RM, Ekström K, El Andaloussi S, Elie-Caille C (2018). Minimal information for studies of extracellular vesicles 2018 (MISEV2018): a position statement of the international society for extracellular vesicles and update of the MISEV2014 guidelines. J Extracell Vesicles.

[25] van Niel G, D'Angelo G, Raposo G (2018). Shedding light on the cell biology of extracellular vesicles. Nat Rev Mol Cell Biol.

[26] Harding C, Heuser J, Stahl P (1984). Endocytosis and intracellular processing of transferrin and colloidal gold-transferrin in rat reticulocytes: demonstration of a pathway for receptor shedding. Eur J Cell Biol.

[27] Pan BT, Teng K, Wu C, Adam M, Johnstone RM (1985). Electron microscopic evidence for externalization of the transferrin receptor in vesicular form in sheep reticulocytes. J Cell Biol.

[28] Tricarico C, Clancy J, D'Souza-Schorey C (2017). Biology and biogenesis of shed microvesicles. Small GTPases.

[29] Dixson AC, Dawson TR, Di Vizio D, Weaver AM (2023). Context-specific regulation of extracellular vesicle biogenesis and cargo selection. Nat Rev Mol Cell Biol.

[30] Lötvall J, Hill AF, Hochberg F, Buzás EI, Di Vizio D, Gardiner C, Gho YS, Kurochkin IV, Mathivanan S, Quesenberry P, Sahoo S, Tahara H, Wauben MH, Witwer KW, Théry C (2014). Minimal experimental requirements for definition of extracellular vesicles and their functions: a position statement from the international society for extracellular vesicles. J Extracell Vesicles.

[31] Witwer KW, Goberdhan DC, O'Driscoll L, Théry C, Welsh JA, Blenkiron C, Buzás EI, Di Vizio D, Erdbrügger U, Falcón-Pérez JM, Fu QL, Hill AF, Lenassi M, Lötvall J, Nieuwland R, Ochiya T, Rome S, Sahoo S, Zheng L (2021). Updating MISEV: evolving the minimal requirements for studies of extracellular vesicles. J Extracell Vesicles.

[32] Lazar I, Clement E, Dauvillier S, Milhas D, Ducoux-Petit M, LeGonidec S (2016). Adipocyte exosomes promote melanoma aggressiveness through fatty acid oxidation: a novel mechanism linking obesity and cancer. Cancer Res.

[33] Lin R, Wang S, Zhao RC (2013). Exosomes from human adipose-derived mesenchymal stem cells promote migration through Wnt signaling pathway in a breast cancer cell model. Mol Cell Biochem.

[34] Ramos-Andrade I, Moraes J, Brandão-Costa RM, Vargas da Silva S, de Souza A, da Silva C, Renovato-Martins M, Barja-Fidalgo C (2020). Obese adipose tissue extracellular vesicles raise breast cancer cell malignancy. Endocr Relat Cancer.

[35] Connolly KD, Guschina IA, Yeung V, Clayton A, Draman MS, Von Ruhland C, Ludgate M, James PE, Rees DA (2015). Characterisation of adipocyte-derived extracellular vesicles released pre- and post-adipogenesis. J Extracell Vesicles.

[36] Camino T, Lago-Baameiro N, Bravo SB, Sueiro A, Couto I, Santos F, Baltar J, Casanueva FF, Pardo M (2020). Vesicles shed by pathological murine adipocytes spread pathology: characterization and functional role of insulin resistant/hypertrophied adiposomes. Int J Mol Sci.

[37] Camino T, Lago-Baameiro N, Bravo SB, Molares-Vila A, Sueiro A, Couto I, Baltar J, Casanueva EF, Pardo M (2022). Human obese white adipose tissue sheds depot-specific extracellular vesicles and reveals candidate biomarkers for monitoring obesity and its comorbidities. Transl Res.

[38] Camino T, Lago-Baameiro N, Martis-Sueiro A, Couto I, Santos F, Baltar J, Pardo M (2020). Deciphering adipose tissue extracellular vesicles protein cargo and its role in obesity. Int J Mol Sci.

[39] Ruiz-Ojeda FJ, Méndez-Gutiérrez A, Aguilera CM, Plaza-Díaz J (2019). Extracellular matrix remodeling of adipose tissue in obesity and metabolic diseases. Int J Mol Sci.

[40] Durcin M, Fleury A, Taillebois E, Hilairet G, Krupova Z, Henry C, Truchet S, Trötzmüller M, Köfeler H, Mabilleau G, Hue O, Andriantsitohaina R, Martin P, Le Lay S (2017). Characterisation of adipocyte-derived extracellular vesicle subtypes identifies distinct protein and lipid signatures for large and small extracellular vesicles. J Extracell Vesicles.

[41] Magoling B, Wu AY, Chen YJ, Wong WW, Chuo ST, Huang HC, Sung YC, Hsieh HT, Huang P, Lee KZ, Huang KW, Chen RH, Chen Y, Lai CP (2023). Membrane protein modification modulates big and small extracellular vesicle biodistribution and tumorigenic potential in breast cancers in vivo. Adv Mater.

[42] Garcia-Martin R, Brandao BB, Thomou T, Altindis E, Kahn CR (2022). Tissue differences in the exosomal/small extracellular vesicle proteome and their potential as indicators of altered tissue metabolism. Cell Rep.

[43] Wu D, Yan J, Shen X, Sun Y, Thulin M, Cai Y, Wik L, Shen Q, Oelrich J, Qian X, Dubois KL, Ronquist KG, Nilsson M, Landegren U, Kamali-Moghaddam M (2019). Profiling surface proteins on individual exosomes using a proximity barcoding assay. Nat Commun.

[44] Weng Z, Zhang B, Wu C, Yu F, Han B, Li B, Li L (2021). Therapeutic roles of mesenchymal stem cell-derived extracellular vesicles in cancer. J Hematol Oncol.

[45] Kosaka N, Kogure A, Yamamoto T, Urabe F, Usuba W, Prieto-Vila M, Ochiya T (2019). Exploiting the message from cancer: the diagnostic value of extracellular vesicles for clinical applications. Exp Mol Med.

[46] Li Y, Talbot CL, Chaurasia B (2020). Ceramides in adipose tissue. Front Endocrinol (Lausanne).

[47] Kim JI, Huh JY, Sohn JH, Choe SS, Lee YS, Lim CY, Jo A, Park SB, Han W, Kim JB (2015). Lipid-overloaded enlarged adipocytes provoke insulin resistance independent of inflammation. Mol Cell Biol.

[48] Giordano C, Gelsomino L, Barone I, Panza S, Augimeri G, Bonofiglio D (2019). Leptin modulates exosome biogenesis in breast cancer cells: an additional mechanism in cell-to-cell communication. J Clin Med.

[49] Gelsomino L, Barone I, Caruso A, Giordano F, Brindisi M, Morello G, Accattatis FM, Panza S, Cappello AR, Bonofiglio D, Andò S, Catalano S, Giordano C (2022). Proteomic profiling of extracellular vesicles released by leptin-treated breast cancer cells: a potential role in cancer metabolism. Int J Mol Sci.

[50] Giordano C, Vizza D, Panza S, Barone I, Bonofiglio D, Lanzino M, Sisci D, De Amicis F, Fuqua SA, Catalano S, Andò S (2013). Leptin increases HER2 protein levels through a STAT3-mediated up-regulation of Hsp90 in breast cancer cells. Mol Oncol.

[51] Guay C, Regazzi R (2017). Exosomes as new players in metabolic organ cross-talk. Diabetes Obes Metab.

[52] Saliminejad K, Khorram Khorshid HR, SoleymaniFard S, Ghaffari SH (2019). An overview of microRNAs: biology, functions, therapeutics, and analysis methods. J Cell Physiol.

[53] Thomou T, Mori MA, Dreyfuss JM, Konishi M, Sakaguchi M, Wolfrum C, Rao TN, Winnay JN, Garcia-Martin R, Grinspoon SK, Gorden P, Kahn CR (2017). Adipose-derived circulating miRNAs regulate gene expression in other tissues. Nature.

[54] Karolina DS, Tavintharan S, Armugam A, Sepramaniam S, Pek SL, Wong MT (2012). Circulating miRNA profiles in patients with metabolic syndrome. J Clin Endocrinol Metab.

[55] Hubal MJ, Nadler EP, Ferrante SC, Barberio MD, Suh JH, Wang J (2017). Circulating adipocyte-derived exosomal microRNAs associated with decreased insulin resistance after gastric bypass. Obesity (Silver Spring).

[56] Guglielmotto M, Aragno M, Autelli R, Giliberto L, Novo E, Colombatto S, Danni O, Parola M, Smith MA, Perry G, Tamagno E, Tabaton M (2009). The up-regulation of BACE1 mediated by hypoxia and ischemic injury: role of oxidative stress and HIF1alpha. J Neurochem.

[57] Greco S, Gaetano C, Martelli F (2014). HypoxamiR regulation and function in ischemic cardiovascular diseases. Antioxid Redox Signal.

[58] Goossens GH, Blaak EE (2015). Adipose tissue dysfunction and impaired metabolic health in human obesity: a matter of oxygen. Front Endocrinol (Lausanne).

[59] Lee JW, Ko J, Ju C, Eltzschig HK (2019). Hypoxia signaling in human diseases and therapeutic targets. Exp Mol Med.

[60] Rapisarda A, Melillo G (2012). Overcoming disappointing results with antiangiogenic therapy by targeting hypoxia. Nat Rev Clin Oncol.

[61] Jain RK (2014). Antiangiogenesis strategies revisited: from starving tumors to alleviating hypoxia. Cancer Cell.

[62] Semenza GL (1985). HIF-1: mediator of physiological and pathophysiological responses to hypoxia. J Appl Physiol.

[63] LaGory EL, Giaccia AJ (2016). The ever-expanding role of HIF in tumour and stromal biology. Nat Cell Biol.

[64] Pugh CW, Ratcliffe PJ (2003). Regulation of angiogenesis by hypoxia: role of the HIF system. Nat Med.

[65] Rius J, Guma M, Schachtrup C, Akassoglou K, Zinkernagel AS, Nizet V, Johnson RS, Haddad GG, Karin M (2008). NF-kappaB links innate immunity to the hypoxic response through transcriptional regulation of HIF-1alpha. Nature.

[66] Hudson CC, Liu M, Chiang GG, Otterness DM, Loomis DC, Kaper F, Giaccia AJ, Abraham RT (2002). Regulation of hypoxia-inducible factor 1alpha expression and function by the mammalian target of rapamycin. Mol Cell Biol.

[67] Noman MZ, Buart S, Van Pelt J, Richon C, Hasmim M, Leleu N, Suchorska WM, Jalil A, Lecluse Y, El Hage F, Giuliani M, Pichon C, Azzarone B, Mazure N, Romero P, Mami-Chouaib F, Chouaib S (2009). The cooperative induction of hypoxia-inducible factor-1 alpha and STAT3 during hypoxia induced an impairment of tumor susceptibility to CTL-mediated cell lysis. J Immunol.

[68] Möller A, Lobb RJ (2020). The evolving translational potential of small extracellular vesicles in cancer. Nat Rev Cancer.

[69] Kalluri R, LeBleu VS (2020). The biology, function, and biomedical applications of exosomes. Science.

[70] Li J, Yuan H, Xu H, Zhao H, Xiong N (2020). Hypoxic cancer-secreted exosomal miR-182-5p promotes glioblastoma angiogenesis by targeting Kruppel-like factor 2 and 4. Mol Cancer Res.

[71] King HW, Michael MZ, Gleadle JM (2012). Hypoxic enhancement of exosome release by breast cancer cells. BMC Cancer.

[72] Jung KO, Jo H, Yu JH, Gambhir SS, Pratx G (2018). Development and MPI tracking of novel hypoxia-targeted theranostic exosomes. Biomaterials.

[73] Matsuura Y, Wada H, Eguchi H, Gotoh K, Kobayashi S, Kinoshita M, Kubo M, Hayashi K, Iwagami Y, Yamada D, Asaoka T, Noda T, Kawamoto K, Takeda Y, Tanemura M, Umeshita K, Doki Y, Mori M (2019). Exosomal miR-155 derived from hepatocellular carcinoma cells under hypoxia promotes angiogenesis in endothelial cells. Dig Dis Sci.

[74] Patton MC, Zubair H, Khan MA, Singh S, Singh AP (2020). Hypoxia alters the release and size distribution of extracellular vesicles in pancreatic cancer cells to support their adaptive survival. J Cell Biochem.

[75] Xia X, Wang S, Ni B, Xing S, Cao H, Zhang Z, Yu F, Zhao E, Zhao G (2020). Hypoxic gastric cancer-derived exosomes promote progression and metastasis via MiR-301a-3p/PHD3/HIF-1α positive feedback loop. Oncogene.

[76] Ren R, Sun H, Ma C, Liu J, Wang H (2019). Colon cancer cells secrete exosomes to promote self-proliferation by shortening mitosis duration and activation of STAT3 in a hypoxic environment. Cell Biosci.

[77] Wang Y, Yin K, Tian J, Xia X, Ma J, Tang X, Xu H, Wang S (2019). Granulocytic myeloid-derived suppressor cells promote the stemness of colorectal cancer cells through exosomal S100A9. Adv Sci (Weinh).

[78] Ramteke A, Ting H, Agarwal C, Mateen S, Somasagara R, Hussain A, Graner M, Frederick B, Agarwal R, Deep G (2015). Exosomes secreted under hypoxia enhance invasiveness and stemness of prostate cancer cells by targeting adherens junction molecules. Mol Carcinog.

[79] Zhu LP, Tian T, Wang JY, He JN, Chen T, Pan M, Xu L, Zhang HX, Qiu XT, Li CC, Wang KK, Shen H, Zhang GG, Bai YP (2018). Hypoxia-elicited mesenchymal stem cell-derived exosomes facilitates cardiac repair through miR-125b-mediated prevention of cell death in myocardial infarction. Theranostics.

[80] Liu W, Li L, Rong Y, Qian D, Chen J, Zhou Z, Luo Y, Jiang D, Cheng L, Zhao S, Kong F, Wang J, Zhou Z, Xu T, Gong F, Huang Y, Gu C, Zhao X, Bai J, Wang F, Zhao W, Zhang L, Li X, Yin G, Fan J, Cai W (2020). Hypoxic mesenchymal stem cell-derived exosomes promote bone fracture healing by the transfer of miR-126. Acta Biomater.

[81] Wang T, Gilkes DM, Takano N, Xiang L, Luo W, Bishop CJ, Chaturvedi P, Green JJ, Semenza GL (2014). Hypoxia-inducible factors and RAB22A mediate formation of microvesicles that stimulate breast cancer invasion and metastasis. Proc Natl Acad Sci USA.

[82] Zhang L, Liu H, Xu K, Ling Z, Huang Y, Hu Q, Lu K, Liu C, Wang Y, Liu N, Zhang X, Xu B, Wu J, Chen S, Zhang G, Chen M (2019). Hypoxia preconditioned renal tubular epithelial cell-derived extracellular vesicles alleviate renal ischaemia–reperfusion injury mediated by the HIF-1α/Rab22 pathway and potentially affected by microRNAs. Int J Biol Sci.

[83] van Niel G, Charrin S, Simoes S, Romao M, Rochin L, Saftig P, Marks MS, Rubinstein E, Raposo G (2011). The tetraspanin CD63 regulates ESCRT-independent and -dependent endosomal sorting during melanogenesis. Dev Cell.

[84] Namazi H, Mohit E, Namazi I, Rajabi S, Samadian A, Hajizadeh-Saffar E, Aghdami N, Baharvand H (2018). Exosomes secreted by hypoxic cardiosphere-derived cells enhance tube formation and increase pro-angiogenic miRNA. J Cell Biochem.

[85] Zhang J, Ma J, Long K, Qiu W, Wang Y, Hu Z, Liu C, Luo Y, Jiang A, Jin L, Tang Q, Wang X, Li X, Li M (2017). Overexpression of exosomal cardioprotective miRNAs mitigates hypoxia-induced H9c2 cells apoptosis. Int J Mol Sci.

[86] Hsu YL, Hung JY, Chang WA, Lin YS, Pan YC, Tsai PH, Wu CY, Kuo PL (2017). Hypoxic lung cancer-secreted exosomal miR-23a increased angiogenesis and vascular permeability by targeting prolyl hydroxylase and tight junction protein ZO-1. Oncogene.

[87] Umezu T, Tadokoro H, Azuma K, Yoshizawa S, Ohyashiki K, Ohyashiki JH (2014). Exosomal miR-135b shed from hypoxic multiple myeloma cells enhances angiogenesis by targeting factor-inhibiting HIF-1. Blood.

[88] Bister N, Pistono C, Huremagic B, Jolkkonen J, Giugno R, Malm T (2020). Hypoxia and extracellular vesicles: a review on methods, vesicular cargo and functions. J Extracell Vesicles.

[89] Zeng Z, Zhao Y, Chen Q, Zhu S, Niu Y, Ye Z, Hu P, Chen D, Xu P, Chen J, Hu C, Hu Y, Xu F, Tang J, Wang F, Han S, Huang M, Wang C, Zhao G (2021). Hypoxic exosomal HIF-1α-stabilizing circZNF91 promotes chemoresistance of normoxic pancreatic cancer cells via enhancing glycolysis. Oncogene.

[90] Yang K, Zhang J, Bao C (2021). Exosomal circEIF3K from cancer-associated fibroblast promotes colorectal cancer (CRC) progression via miR-214/PD-L1 axis. BMC Cancer.

[91] Zhang C, Wang H, Li J, Ma L (2021). Circular RNA involvement in the protective effect of human umbilical cord mesenchymal stromal cell-derived extracellular vesicles against hypoxia/reoxygenation injury in cardiac cells. Front Cardiovasc Med.

[92] Wang Y, Zhao R, Liu W, Wang Z, Rong J, Long X, Liu Z, Ge J, Shi B (2019). Exosomal circHIPK3 released from hypoxia-pretreated cardiomyocytes regulates oxidative damage in cardiac microvascular endothelial cells via the miR-29a/IGF-1 pathway. Oxid Med Cell Longev.

[93] Yang H, Zhang H, Yang Y, Wang X, Deng T, Liu R, Ning T, Bai M, Li H, Zhu K, Li J, Fan Q, Ying G, Ba Y (2022). Erratum: Hypoxia induced exosomal circRNA promotes metastasis of colorectal cancer via targeting GEF-H1/RhoA axis. Theranostics.

[94] Kucharzewska P, Christianson HC, Welch JE, Svensson KJ, Fredlund E, Ringnér M, Mörgelin M, Bourseau-Guilmain E, Bengzon J, Belting M (2013). Exosomes reflect the hypoxic status of glioma cells and mediate hypoxia-dependent activation of vascular cells during tumor development. Proc Natl Acad Sci USA.

[95] Kenneweg F, Bang C, Xiao K, Boulanger CM, Loyer X, Mazlan S, Schroen B, Hermans-Beijnsberger S, Foinquinos A, Hirt MN, Eschenhagen T, Funcke S, Stojanovic S, Genschel C, Schimmel K, Just A, Pfanne A, Scherf K, Dehmel S, Raemon-Buettner SM, Fiedler J, Thum T (2019). Long noncoding RNA-enriched vesicles secreted by hypoxic cardiomyocytes drive cardiac fibrosis. Mol Ther Nucleic Acids.

[96] Trayhurn P (2022). Adipokines: inflammation and the pleiotropic role of white adipose tissue. Br J Nutr.

[97] Sano S, Izumi Y, Yamaguchi T, Yamazaki T, Tanaka M, Shiota M, Osada-Oka M, Nakamura Y, Wei M, Wanibuchi H, Iwao H, Yoshiyama M (2014). Lipid synthesis is promoted by hypoxic adipocyte-derived exosomes in 3T3-L1 cells. Biochem Biophys Res Commun.

[98] Trayhurn P (2014). Hypoxia and adipocyte physiology: implications for adipose tissue dysfunction in obesity. Annu Rev Nutr.

[99] Matafome P, Rodrigues T, Seica R (2015). Glycation and hypoxia: two key factors for adipose tissue dysfunction. Curr Med Chem.

[100] Arendt LM, McCready J, Keller PJ, Baker DD, Naber SP, Seewaldt V, Kuperwasser C (2013). Obesity promotes breast cancer by CCL2-mediated macrophage recruitment and angiogenesis. Cancer Res.

[101] Lapeire L, Hendrix A, Lambein K, Van Bockstal M, Braems G, Van Den Broecke R, Limame R, Mestdagh P, Vandesompele J, Vanhove C, Maynard D, Lehuédé C, Muller C, Valet P, Gespach CP, Bracke M, Cocquyt V, Denys H, De Wever O (2014). Cancer-associated adipose tissue promotes breast cancer progression by paracrine oncostatin M and Jak/STAT3 signaling. Cancer Res.

[102] Hoy AJ, Balaban S, Saunders DN (2017). Adipocyte-tumor cell metabolic crosstalk in breast cancer. Trends Mol Med.

[103] Helmlinger G, Yuan F, Dellian M, Jain RK (1997). Interstitial pH and pO2 gradients in solid tumors in vivo: high-resolution measurements reveal a lack of correlation. Nat Med.

[104] Quail DF, Dannenberg AJ (2019). The obese adipose tissue microenvironment in cancer development and progression. Nat Rev Endocrinol.

[105] Iyengar NM, Gucalp A, Dannenberg AJ, Hudis CA (2016). Obesity and cancer mechanisms: tumor microenvironment and inflammation. J Clin Oncol.

[106] Ye J, Gao Z, Yin J, He Q (2007). Hypoxia is a potential risk factor for chronic inflammation and adiponectin reduction in adipose tissue of ob/ob and dietary obese mice. Am J Physiol Endocrinol Metab.

[107] He Q, Gao Z, Yin J, Zhang J, Yun Z, Ye J (2011). Regulation of HIF-1α activity in adipose tissue by obesity-associated factors: adipogenesis, insulin, and hypoxia. Am J Physiol Endocrinol Metab.

[108] Kong JY, Rabkin SW (2000). Palmitate-induced apoptosis in cardiomyocytes is mediated through alterations in mitochondria: prevention by cyclosporin A. Biochim Biophys Acta.

[109] Norouzirad R, González-Muniesa P, Ghasemi A (2017). Hypoxia in obesity and diabetes: potential therapeutic effects of hyperoxia and nitrate. Oxid Med Cell Longev.

[110] Seo JB, Riopel M, Cabrales P, Huh JY, Bandyopadhyay GK, Andreyev AY, Murphy AN, Beeman SC, Smith GI, Klein S, Lee YS, Olefsky JM (2019). Knockdown of Ant2 reduces adipocyte hypoxia and improves insulin resistance in obesity. Nat Metab.

[111] Ozmen F, Ozmen MM, Gelecek S, Bilgic İ, Moran M, Sahin TT (2016). STEAP4 and HIF-1α gene expressions in visceral and subcutaneous adipose tissue of the morbidly obese patients. Mol Immunol.

[112] Warbrick I, Rabkin SW (2019). Hypoxia-inducible factor 1-alpha (HIF-1α) as a factor mediating the relationship between obesity and heart failure with preserved ejection fraction. Obes Rev.

[113] Wang J, Wu Y, Guo J, Fei X, Yu L, Ma S (2017). Adipocyte-derived exosomes promote lung cancer metastasis by increasing MMP9 activity via transferring MMP3 to lung cancer cells. Oncotarget.

[114] Liu Y, Tan J, Ou S, Chen J, Chen L (2019). Adipose-derived exosomes deliver miR-23a/b to regulate tumor growth in hepatocellular cancer by targeting the VHL/HIF axis. J Physiol Biochem.

[115] Hanahan D, Weinberg RA (2000). The hallmarks of cancer. Cell.

[116] Garrett WS (2015). Cancer and the microbiota. Science.

[117] Zhong Z, Sanchez-Lopez E, Karin M (2016). Autophagy, inflammation, and immunity: a troika governing cancer and its treatment. Cell.

[118] Ringel AE, Drijvers JM, Baker GJ, Catozzi A, García-Cañaveras JC, Gassaway BM, Miller BC, Juneja VR, Nguyen TH, Joshi S, Yao CH, Yoon H, Sage PT, LaFleur MW, Trombley JD, Jacobson CA, Maliga Z, Gygi SP, Sorger PK, Rabinowitz JD, Sharpe AH, Haigis MC (2020). Obesity shapes metabolism in the tumor microenvironment to suppress anti-tumor immunity. Cell.

[119] Ying W, Riopel M, Bandyopadhyay G, Dong Y, Birmingham A, Seo JB, Ofrecio JM, Wollam J, Hernandez-Carretero A, Fu W, Li P, Olefsky JM (2017). Adipose tissue macrophage-derived exosomal miRNAs can modulate in vivo and in vitro insulin sensitivity. Cell.

[120] Mattiske S, Suetani RJ, Neilsen PM, Callen DF (2012). The oncogenic role of miR-155 in breast cancer. Cancer Epidemiol Biomark Prev.

[121] Zhang CM, Zhao J, Deng HY (2013). MiR-155 promotes proliferation of human breast cancer MCF-7 cells through targeting tumor protein 53-induced nuclear protein 1. J Biomed Sci.

[122] Zimta AA, Tigu AB, Muntean M, Cenariu D, Slaby O, Berindan-Neagoe I (2019). Molecular links between central obesity and breast cancer. Int J Mol Sci.

[123] Zhang Q, Deng T, Zhang H, Zuo D, Zhu Q, Bai M, Liu R, Ning T, Zhang L, Yu Z, Zhang H, Ba Y (2022). Adipocyte-derived exosomal MTTP suppresses ferroptosis and promotes chemoresistance in colorectal cancer. Adv Sci (Weinh).

[124] Peng H, Chiu TY, Liang YJ, Lee CJ, Liu CS, Suen CS, Yen JJ, Chen HT, Hwang MJ, Hussain MM, Yang HC, Yang-Yen HF (2021). PRAP1 is a novel lipid-binding protein that promotes lipid absorption by facilitating MTTP-mediated lipid transport. J Biol Chem.

[125] Wetterau JR, Zilversmit DB (1985). Purification and characterization of microsomal triglyceride and cholesteryl ester transfer protein from bovine liver microsomes. Chem Phys Lipids.

[126] He L, Zhu C, Jia J, Hao XY, Yu XY, Liu XY, Shu MG (2020). ADSC-Exos containing MALAT1 promotes wound healing by targeting miR-124 through activating Wnt/β-catenin pathway. Biosci Rep.

[127] Annett S, Moore G, Robson T (2020). Obesity and cancer metastasis: molecular and translational perspective. Cancers (Basel).

[128] Wu Q, Li B, Li Z, Li J, Sun S, Sun S (2019). Cancer-associated adipocytes: key players in breast cancer progression. J Hematol Oncol.

[129] Senger DR, Davis GE (2011). Angiogenesis. Cold Spring Harb Perspect Biol.

[130] Goveia J, Stapor P, Carmeliet P (2014). Principles of targeting endothelial cell metabolism to treat angiogenesis and endothelial cell dysfunction in disease. EMBO Mol Med.

[131] McIntyre A, Harris AL (2015). Metabolic and hypoxic adaptation to anti-angiogenic therapy: a target for induced essentiality. EMBO Mol Med.

[132] Harjes U, Bensaad K, Harris AL (2012). Endothelial cell metabolism and implications for cancer therapy. Br J Cancer.

[133] Kapur SK, Katz AJ (2013). Review of the adipose derived stem cell secretome. Biochimie.

[134] Gangadaran P, Rajendran RL, Oh JM, Oh EJ, Hong CM, Chung HY, Lee J, Ahn BC (2021). Identification of angiogenic cargo in extracellular vesicles secreted from human adipose tissue-derived stem cells and induction of angiogenesis in vitro and in vivo. Pharmaceutics.

[135] Wang X, Wang H, Cao J, Ye C (2018). Exosomes from adipose-derived stem cells promotes VEGF-C-dependent lymphangiogenesis by regulating miRNA-132/TGF-β pathway. Cell Physiol Biochem.

[136] Yamashita M (2015). Lymphangiogenesis and lesion heterogeneity in interstitial lung diseases. Clin Med Insights Circ Respir Pulm Med.

[137] Deng HT, Liu HL, Zhai BB, Zhang K, Xu GC, Peng XM, Zhang QZ, Li LY (2017). Vascular endothelial growth factor suppresses TNFSF15 production in endothelial cells by stimulating miR-31 and miR-20a expression via activation of Akt and Erk signals. FEBS Open Bio.

[138] Kang T, Jones TM, Naddell C, Bacanamwo M, Calvert JW, Thompson WE, Bond VC, Chen YE, Liu D (2016). Adipose-derived stem cells induce angiogenesis via microvesicle transport of miRNA-31. Stem Cells Transl Med.

[139] Demirkan B (2013). The roles of epithelial-to-mesenchymal transition (EMT) and mesenchymal-to-epithelial transition (MET) in breast cancer bone metastasis: potential targets for prevention and treatment. J Clin Med.

[140] Wu Y, Sarkissyan M, Vadgama JV (2016). Epithelial–mesenchymal transition and breast cancer. J Clin Med.

[141] Khanh VC, Fukushige M, Moriguchi K, Yamashita T, Osaka M, Hiramatsu Y, Ohneda O (2020). Type 2 diabetes mellitus induced paracrine effects on breast cancer metastasis through extracellular vesicles derived from human mesenchymal stem cells. Stem Cells Dev.

[142] Yang H, Zhang H, Yang Y, Wang X, Deng T, Liu R, Ning T, Bai M, Li H, Zhu K, Li J, Fan Q, Ying G, Ba Y (2020). Hypoxia induced exosomal circRNA promotes metastasis of colorectal cancer via targeting GEF-H1/RhoA axis. Theranostics.

[143] Xue M, Chen W, Xiang A, Wang R, Chen H, Pan J, Pang H, An H, Wang X, Hou H, Li X (2017). Hypoxic exosomes facilitate bladder tumor growth and development through transferring long non-coding RNA-UCA1. Mol Cancer.

[144] Sun X, Casbas-Hernandez P, Bigelow C, Makowski L, Joseph Jerry D, Smith Schneider S, Troester MA (2012). Normal breast tissue of obese women is enriched for macrophage markers and macrophage-associated gene expression. Breast Cancer Res Treat.

[145] Nieman KM, Kenny HA, Penicka CV, Ladanyi A, Buell-Gutbrod R, Zillhardt MR, Romero IL, Carey MS, Mills GB, Hotamisligil GS, Yamada SD, Peter ME, Gwin K, Lengyel E (2011). Adipocytes promote ovarian cancer metastasis and provide energy for rapid tumor growth. Nat Med.

[146] Zhao H, Shang Q, Pan Z, Bai Y, Li Z, Zhang H, Zhang Q, Guo C, Zhang L, Wang Q (2018). Exosomes from adipose-derived stem cells attenuate adipose inflammation and obesity through polarizing M2 macrophages and beiging in white adipose tissue. Diabetes.

[147] Boutilier AJ, Elsawa SF (2021). Macrophage polarization states in the tumor microenvironment. Int J Mol Sci.

[148] Biswas SK, Allavena P, Mantovani A (2013). Tumor-associated macrophages: functional diversity, clinical significance, and open questions. Semin Immunopathol.

[149] Pardoll DM (2012). The blockade of immune checkpoints in cancer immunotherapy. Nat Rev Cancer.

[150] Li Z, Zhang C, Du JX, Zhao J, Shi MT, Jin MW, Liu H (2020). Adipocytes promote tumor progression and induce PD-L1 expression via TNF-α/IL-6 signaling. Cancer Cell Int.

[151] Li L, Cao B, Liang X, Lu S, Luo H, Wang Z, Wang S, Jiang J, Lang J, Zhu G (2019). Microenvironmental oxygen pressure orchestrates an anti- and pro-tumoral γδ T cell equilibrium via tumor-derived exosomes. Oncogene.

[152] Warburg O (1956). On the origin of cancer cells. Science.

[153] Nieman KM, Romero IL, Van Houten B, Lengyel E (1831). Adipose tissue and adipocytes support tumorigenesis and metastasis. Biochim Biophys Acta.

[154] Lengyel E, Makowski L, DiGiovanni J, Kolonin MG (2018). Cancer as a matter of fat: the crosstalk between adipose tissue and tumors, trends. Cancer.

[155] Duman C, Yaqubi K, Hoffmann A, Acikgöz AA, Korshunov A, Bendszus M, Herold-Mende C, Liu HK, Alfonso J (2019). Acyl-CoA-binding protein drives glioblastoma tumorigenesis by sustaining fatty acid oxidation. Cell Metab.

[156] Nomura DK, Long JZ, Niessen S, Hoover HS, Ng SW, Cravatt BF (2010). Monoacylglycerol lipase regulates a fatty acid network that promotes cancer pathogenesis. Cell.

[157] Robado de Lope L, Alcíbar OL, AmorLópez A, Hergueta-Redondo M, Peinado H (2018). Tumour-adipose tissue crosstalk: fuelling tumour metastasis by extracellular vesicles. Philos Trans R Soc Lond B Biol Sci.

[158] Feng S, Lou K, Luo C, Zou J, Zou X, Zhang G (2022). Obesity-related cross-talk between prostate cancer and peripheral fat: potential role of exosomes. Cancers (Basel).

[159] Das A, Mohan V, Krishnaswamy VR, Solomonov I, Sagi I (2019). Exosomes as a storehouse of tissue remodeling proteases and mediators of cancer progression. Cancer Metastasis Rev.

[160] Vallabhaneni KC, Penfornis P, Dhule S, Guillonneau F, Adams KV, Mo YY, Xu R, Liu Y, Watabe K, Vemuri MC, Pochampally R (2015). Extracellular vesicles from bone marrow mesenchymal stem/stromal cells transport tumor regulatory microRNA, proteins, and metabolites. Oncotarget.

[161] Cao Y (2019). Adipocyte and lipid metabolism in cancer drug resistance. J Clin Invest.

[162] Jafari N, Kolla M, Meshulam T, Shafran JS, Qiu Y, Casey AN (2021). Adipocyte-derived exosomes may promote breast cancer progression in type 2 diabetes. Sci Signal.

[163] Milbank E, Dragano N, Vidal-Gómez X, Rivas-Limeres V, Garrido-Gil P, Wertheimer M (2023). Small extracellular vesicle targeting of hypothalamic AMPKα1 promotes weight loss in leptin receptor deficient mice. Metabolism.

[164] Liu S, Benito-Martin A, Pelissier Vatter FA, Hanif SZ, Liu C, Bhardwaj P (2023). Breast adipose tissue-derived extracellular vesicles from women with obesity stimulate mitochondrial-induced dysregulated tumor cell metabolism. bioRxiv.

[165] Kulaj K, Harger A, Bauer M, Caliskan ÖS, Gupta TK, Chiang DM (2023). Adipocyte-derived extracellular vesicles increase insulin secretion through transport of insulinotropic protein cargo. Nat Commun.

[166] Nakamura Y, Taniguchi H, Ikeda M, Bando H, Kato K, Morizane C, Esaki T, Komatsu Y, Kawamoto Y, Takahashi N, Ueno M, Kagawa Y, Nishina T, Kato T, Yamamoto Y, Furuse J, Denda T, Kawakami H, Oki E, Nakajima T, Nishida N, Yamaguchi K, Yasui H, Goto M, Matsuhashi N, Ohtsubo K, Yamazaki K, Tsuji A, Okamoto W, Tsuchihara K, Yamanaka T, Miki I, Sakamoto Y, Ichiki H, Hata M, Yamashita R, Ohtsu A, Odegaard JI, Yoshino T (2020). Clinical utility of circulating tumor DNA sequencing in advanced gastrointestinal cancer: SCRUM-Japan GI-SCREEN and GOZILA studies. Nat Med.

[167] Pantel K, Alix-Panabières C (2019). Liquid biopsy and minimal residual disease—latest advances and implications for cure. Nat Rev Clin Oncol.

[168] Mitchell PS, Parkin RK, Kroh EM, Fritz BR, Wyman SK, Pogosova-Agadjanyan EL, Peterson A, Noteboom J, O'Briant KC, Allen A, Lin DW, Urban N, Drescher CW, Knudsen BS, Stirewalt DL, Gentleman R, Vessella RL, Nelson PS, Martin DB, Tewari M (2008). Circulating microRNAs as stable blood-based markers for cancer detection. Proc Natl Acad Sci USA.

[169] Grasedieck S, Schöler N, Bommer M, Niess JH, Tumani H, Rouhi A, Bloehdorn J, Liebisch P, Mertens D, Döhner H, Buske C, Langer C, Kuchenbauer F (2012). Impact of serum storage conditions on microRNA stability. Leukemia.

[170] Selth LA, Tilley WD, Butler LM (2012). Circulating microRNAs: macro-utility as markers of prostate cancer. Endocr Relat Cancer.

[171] Skotland T, Hessvik NP, Sandvig K, Llorente A (2019). Exosomal lipid composition and the role of ether lipids and phosphoinositides in exosome biology. J Lipid Res.

[172] Skotland T, Ekroos K, Kauhanen D, Simolin H, Seierstad T, Berge V, Sandvig K, Llorente A (2017). Molecular lipid species in urinary exosomes as potential prostate cancer biomarkers. Eur J Cancer.

[173] Puhka M, Takatalo M, Nordberg ME, Valkonen S, Nandania J, Aatonen M, Yliperttula M, Laitinen S, Velagapudi V, Mirtti T, Kallioniemi O, Rannikko A, Siljander PR, AfHällström TM (2017). Metabolomic profiling of extracellular vesicles and alternative normalization methods reveal enriched metabolites and strategies to study prostate cancer-related changes. Theranostics.

[174] Zhang Z, Liu X, Yang X, Jiang Y, Li A, Cong J, Li Y, Xie Q, Xu C, Liu D (2023). Identification of faecal extracellular vesicles as novel biomarkers for the non-invasive diagnosis and prognosis of colorectal cancer. J Extracell Vesicles.

[175] Cho S, Yang HC, Rhee WJ (2019). Simultaneous multiplexed detection of exosomal microRNAs and surface proteins for prostate cancer diagnosis. Biosens Bioelectron.

[176] Guo Q, Jiang C (2020). Delivery strategies for macromolecular drugs in cancer therapy. Acta Pharm Sin B.

[177] Li F, Li H, Jin X, Zhang Y, Kang X, Zhang Z, Xu M, Qian Z, Ma Z, Gao X, Zhao L, Wu S, Sun H (2019). Adipose-specific knockdown of Sirt1 results in obesity and insulin resistance by promoting exosomes release. Cell Cycle.

[178] Zhou Y, Tan C (2020). miRNAs in adipocyte-derived extracellular vesicles: multiple roles in development of obesity-associated disease. Front Mol Biosci.

[179] Li T, Zhou X, Wang J, Liu Z, Han S, Wan L, Sun X, Chen H (2020). Adipose-derived mesenchymal stem cells and extracellular vesicles confer antitumor activity in preclinical treatment of breast cancer. Pharmacol Res.

[180] Ramdasi S, Sarang S, Viswanathan C (2015). Potential of mesenchymal stem cell based application in cancer. Int J Hematol Oncol Stem Cell Res.

[181] Chin KY (2016). The spice for joint inflammation: anti-inflammatory role of curcumin in treating osteoarthritis. Drug Des Dev Ther.

[182] Sun D, Zhuang X, Xiang X, Liu Y, Zhang S, Liu C, Barnes S, Grizzle W, Miller D, Zhang HG (2010). A novel nanoparticle drug delivery system: the anti-inflammatory activity of curcumin is enhanced when encapsulated in exosomes. Mol Ther.

[183] Trotta T, Panaro MA, Prifti E, Porro C (2019). Modulation of biological activities in glioblastoma mediated by curcumin. Nutr Cancer.

[184] Yang Z, Xie J, Zhu J, Kang C, Chiang C, Wang X, Wang X, Kuang T, Chen F, Chen Z, Zhang A, Yu B, Lee RJ, Teng L, Lee LJ (2016). Functional exosome-mimic for delivery of siRNA to cancer: in vitro and in vivo evaluation. J Control Release.

[185] Kranz LM, Diken M, Haas H, Kreiter S, Loquai C, Reuter KC, Meng M, Fritz D, Vascotto F, Hefesha H, Grunwitz C, Vormehr M, Hüsemann Y, Selmi A, Kuhn AN, Buck J, Derhovanessian E, Rae R, Attig S, Diekmann J, Jabulowsky RA, Heesch S, Hassel J, Langguth P, Grabbe S, Huber C, Türeci Ö, Sahin U (2016). Systemic RNA delivery to dendritic cells exploits antiviral defence for cancer immunotherapy. Nature.

[186] Mizrahy S, Hazan-Halevy I, Landesman-Milo D, Ng BD, Peer D (2017). Advanced strategies in immune modulation of cancer using lipid-based nanoparticles. Front Immunol.

[187] Oberli MA, Reichmuth AM, Dorkin JR, Mitchell MJ, Fenton OS, Jaklenec A, Anderson DG, Langer R, Blankschtein D (2017). Lipid nanoparticle assisted mRNA delivery for potent cancer immunotherapy. Nano Lett.

[188] Bruno S, Collino F, Deregibus MC, Grange C, Tetta C, Camussi G (2013). Microvesicles derived from human bone marrow mesenchymal stem cells inhibit tumor growth. Stem Cells Dev.

[189] Belmar-Lopez C, Mendoza G, Oberg D, Burnet J, Simon C, Cervello I, Iglesias M, Ramirez JC, Lopez-Larrubia P, Quintanilla M, Martin-Duque P (2013). Tissue-derived mesenchymal stromal cells used as vehicles for anti-tumor therapy exert different in vivo effects on migration capacity and tumor growth. BMC Med.

[190] Rani S, Ryan AE, Griffin MD, Ritter T (2015). Mesenchymal stem cell-derived extracellular vesicles: toward cell-free therapeutic applications. Mol Ther.

[191] Zhang Y, Mei H, Chang X, Chen F, Zhu Y, Han X (2016). Adipocyte-derived microvesicles from obese mice induce M1 macrophage phenotype through secreted miR-155. J Mol Cell Biol.

[192] Togliatto G, Dentelli P, Gili M, Gallo S, Deregibus C, Biglieri E (2016). Obesity reduces the pro-angiogenic potential of adipose tissue stem cell-derived extracellular vesicles (EVs) by impairing miR-126 content: impact on clinical applications. Int J Obes (Lond).

[193] Harrington LS, Findlay GM, Lamb RF (2005). Restraining PI3K: mTOR signalling goes back to the membrane. Trends Biochem Sci.

[194] Belfiore A, Malaguarnera R (2011). Insulin receptor and cancer. Endocr Relat Cancer.

[195] Lohmann AE, Goodwin PJ, Chlebowski RT, Pan K, Stambolic V, Dowling RJ (2016). Association of obesity-related metabolic disruptions with cancer risk and outcome. J Clin Oncol.

[196] Mleczko J, Ortega FJ, Falcon-Perez JM, Wabitsch M, Fernandez-Real JM, Mora S (2018). Extracellular vesicles from hypoxic adipocytes and obese subjects reduce insulin-stimulated glucose uptake. Mol Nutr Food Res.

[197] Yan C, Tian X, Li J, Liu D, Ye D, Xie Z (2021). A high-fat diet attenuates AMPK α1 in adipocytes to induce exosome shedding and nonalcoholic fatty liver development in vivo. Diabetes.

[198] Yang E, Wang X, Gong Z, Yu M, Wu H, Zhang D (2020). Exosome-mediated metabolic reprogramming: the emerging role in tumor microenvironment remodeling and its influence on cancer progression. Signal Transduct Target Ther.

[199] Dirat B, Bochet L, Dabek M, Daviaud D, Dauvillier S, Majed B (2011). Cancer-associated adipocytes exhibit an activated phenotype and contribute to breast cancer invasion. Cancer Res.

[200] Argilés JM, Busquets S, Stemmler B, López-Soriano FJ (2014). Cancer cachexia: understanding the molecular basis. Nat Rev Cancer.

[201] Jeurissen S, Vergauwen G, Van Deun J, Lapeire L, Depoorter V, Miinalainen I, Sormunen R, Van den Broecke R, Braems G, Cocquyt V, Denys H, Hendrix A (2017). The isolation of morphologically intact and biologically active extracellular vesicles from the secretome of cancer-associated adipose tissue. Cell Adh Migr.

[202] Gernapudi R, Yao Y, Zhang Y, Wolfson B, Roy S, Duru N (2015). Targeting exosomes from preadipocytes inhibits preadipocyte to cancer stem cell signaling in early-stage breast cancer. Breast Cancer Res Treat.

[203] Di Vizio D, Morello M, Dudley AC, Schow PW, Adam RM, Morley S (2012). Large oncosomes in human prostate cancer tissues and in the circulation of mice with metastatic disease. Am J Pathol.

[204] Bian X, Xiao YT, Wu T, Yao M, Du L, Ren S (2019). Microvesicles and chemokines in tumor microenvironment: mediators of intercellular communications in tumor progression. Mol Cancer.

[205] Zhou M, Li YJ, Tang YC, Hao XY, Xu WJ, Xiang DX (2022). Apoptotic bodies for advanced drug delivery and therapy. J Control Release.

[206] Zhao X, Lei Y, Zheng J, Peng J, Li Y, Yu L (2019). Identification of markers for migrasome detection. Cell Discov.

[207] Jiao H, Jiang D, Hu X, Du W, Ji L, Yang Y (2021). Mitocytosis, a migrasome-mediated mitochondrial quality-control process. Cell.

[208] Zhang H, Freitas D, Kim HS, Fabijanic K, Li Z, Chen H (2018). Identification of distinct nanoparticles and subsets of extracellular vesicles by asymmetric flow field-flow fractionation. Nat Cell Biol.

[209] Zhang Q, Jeppesen DK, Higginbotham JN, Graves-Deal R, Trinh VQ, Ramirez MA (2021). Supermeres are functional extracellular nanoparticles replete with disease biomarkers and therapeutic targets. Nat Cell Biol.

[210] Ferrante SC, Nadler EP, Pillai DK, Hubal MJ, Wang Z, Wang JM, Gordish-Dressman H, Koeck E, Sevilla S, Wiles AA, Freishtat RJ (2015). Adipocyte-derived exosomal miRNAs: a novel mechanism for obesity-related disease. Pediatr Res.

[211] Zhuang G, Meng C, Guo X, Cheruku PS, Shi L, Xu H, Li H, Wang G, Evans AR, Safe S, Wu C, Zhou B (2012). A novel regulator of macrophage activation: miR-223 in obesity-associated adipose tissue inflammation. Circulation.

[212] Pan Y, Hui X, Hoo R, Ye D, Chan C, Feng T, Wang Y, Lam K, Xu A (2019). Adipocyte-secreted exosomal microRNA-34a inhibits M2 macrophage polarization to promote obesity-induced adipose inflammation. J Clin Invest.

[213] Dang SY, Leng Y, Wang ZX, Xiao X, Zhang X, Wen T, Gong HZ, Hong A, Ma Y (2019). Exosomal transfer of obesity adipose tissue for decreased miR-141-3p mediate insulin resistance of hepatocytes. Int J Biol Sci.

[214] Li D, Song H, Shuo L, Wang L, Xie P, Li W, Liu J, Tong Y, Zhang CY, Jiang X, Li J, Zhang Y (2020). Gonadal white adipose tissue-derived exosomal MiR-222 promotes obesity-associated insulin resistance. Aging (Albany NY).

[215] Yu Y, Du H, Wei S, Feng L, Li J, Yao F, Zhang M, Hatch GM, Chen L (2018). Adipocyte-derived exosomal MiR-27a induces insulin resistance in skeletal muscle through repression of PPARγ. Theranostics.

[216] Li X, Ballantyne LL, Yu Y, Funk CD (2019). Perivascular adipose tissue-derived extracellular vesicle miR-221-3p mediates vascular remodeling. FASEB J.

[217] Gao J, Li X, Wang Y, Cao Y, Yao D, Sun L, Qin L, Qiu H, Zhan X (2020). Adipocyte-derived extracellular vesicles modulate appetite and weight through mTOR signalling in the hypothalamus. Acta Physiol (Oxf).

[218] Wang S, Su X, Xu M, Xiao X, Li X, Li H, Keating A, Zhao RC (2019). Exosomes secreted by mesenchymal stromal/stem cell-derived adipocytes promote breast cancer cell growth via activation of Hippo signaling pathway. Stem Cell Res Ther.

[219] Wu S, Wang Y, Yuan Z, Wang S, Du H, Liu X, Wang Q, Zhu X (2019). Human adipose-derived mesenchymal stem cells promote breast cancer MCF7 cell epithelial-mesenchymal transition by cross interacting with the TGF-β/Smad and PI3K/AKT signaling pathways. Mol Med Rep.

[220] Lou G, Song X, Yang F, Wu S, Wang J, Chen Z, Liu Y (2015). Exosomes derived from miR-122-modified adipose tissue-derived MSCs increase chemosensitivity of hepatocellular carcinoma. J Hematol Oncol.

[221] Quan M, Kuang S (2020). Exosomal secretion of adipose tissue during various physiological states. Pharm Res.

[222] Zhang H, Deng T, Ge S, Liu Y, Bai M, Zhu K, Fan Q, Li J, Ning T, Tian F, Li H, Sun W, Ying G, Ba Y (2019). Exosome circRNA secreted from adipocytes promotes the growth of hepatocellular carcinoma by targeting deubiquitination-related USP7. Oncogene.

[223] An Y, Zhang Z, Shang Y, Jiang X, Dong J, Yu P, Nie Y, Zhao Q (2015). miR-23b-3p regulates the chemoresistance of gastric cancer cells by targeting ATG12 and HMGB2. Cell Death Dis.

[224] Yin H, Qiu X, Shan Y, You B, Xie L, Zhang P, Zhao J, You Y (2021). HIF-1α downregulation of miR-433-3p in adipocyte-derived exosomes contributes to NPC progression via targeting SCD1. Cancer Sci.

[225] Blazquez R, Sanchez-Margallo FM, de la Rosa O, Dalemans W, Alvarez V, Tarazona R, Casado JG (2014). Immunomodulatory potential of human adipose mesenchymal stem cells derived exosomes on in vitro stimulated T cells. Front Immunol.

